# First and Second Reductions in an Aprotic Solvent: Comparing Computational and Experimental One-Electron Reduction Potentials for 345 Quinones

**DOI:** 10.1021/acs.jctc.4c00602

**Published:** 2024-07-06

**Authors:** Sarah Elhajj, Samer Gozem

**Affiliations:** Department of Chemistry, Georgia State University, Atlanta, Georgia 30302, United States

## Abstract

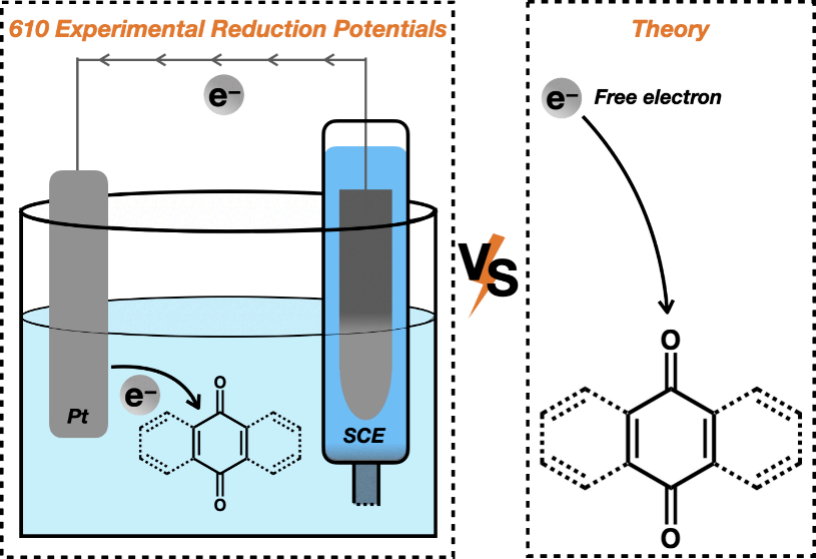

Using reference reduction potentials of quinones recently measured relative to the saturated calomel electrode (SCE) in N,N-dimethylformamide (DMF), we benchmark absolute one-electron reduction potentials computed for 345 Q/Q^•–^ and 265 Q^•–^/Q^2–^ half-reactions using adiabatic electron affinities computed with density functional theory and solvation energies computed with four continuum solvation models: IEF-PCM, C-PCM, COSMO, and SM12. Regression analyses indicate a strong linear correlation between experimental and absolute computed Q/Q^•–^ reduction potentials with Pearson’s correlation coefficient (*r*) between 0.95 and 0.96 and the mean absolute error (MAE) relative to the linear fit between 83.29 and 89.51 mV for different solvation methods when the slope of the regression is constrained to 1. The same analysis for Q^•–^/Q^2–^ gave a linear regression with *r* between 0.74 and 0.90 and MAE between 95.87 and 144.53 mV, respectively. The y-intercept values obtained from the linear regressions are in good agreement with the range of absolute reduction potentials reported in the literature for the SCE but reveal several sources of systematic error. The y-intercepts from Q^•–^/Q^2–^ calculations are lower than those from Q/Q^•–^ by around 320–410 mV for IEF-PCM, C-PCM, and SM12 compared to 210 mV for COSMO. Systematic errors also arise between molecules having different ring sizes (benzoquinones, naphthoquinones, and anthraquinones) and different substituents (titratable vs nontitratable). SCF convergence issues were found to be a source of random error that was slightly reduced by directly optimizing the solute structure in the continuum solvent reaction field. While SM12 MAEs were lower than those of the other solvation models for Q/Q^•–^, SM12 had larger MAEs for Q^•–^/Q^2–^ pointing to a larger error when describing multiply charged anions in DMF. Altogether, the results highlight the advantages of, and further need for, testing computational methods using a large experimental data set that is not skewed (e.g., having more titratable than nontitratable substituents on different parent groups or vice versa) to help further distinguish between sources of random and systematic errors in the calculations.

## Introduction

Electron transfer reactions are ubiquitous processes in redox reactions^1,2^ such as photocatalysis,^3^ photobiocatalysis,^4−6^ electrocatalysis,^7−9^ sensing,^10−12^ energy harvesting,^13,14^ and flow batteries.^15,16^ The fundamental thermodynamic quantity that determines the driving force for redox reactions to occur is the redox potential.^17^ The redox potential, typically measured by cyclic voltammetry when a molecule exhibits a reversible voltammetric wave,^18^ determines the propensity of that molecule to accept or donate electrons.^17,19,20^ Computations provide a distinct route to predicting redox potentials from first principles. Such calculations, when predictive, can be used to simulate redox properties of new materials or to determine potentials for systems under difficult experimental conditions such as for nonreversible redox reactions or highly unstable species.^20,21^

**Scheme 1:**
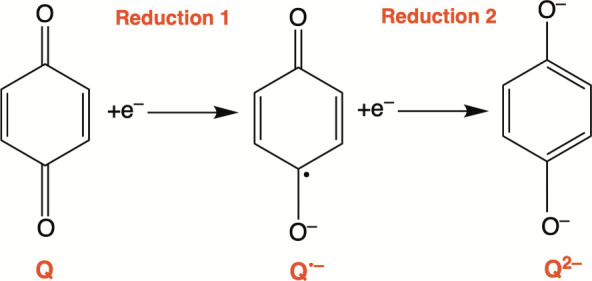
Quinone Reduction in an Aprotic Solvent

Quinones are a class of conjugated redox-active molecules derived from aromatic compounds and containing two carbonyls in a cyclic arrangement.^22^ Due to their redox characteristics, quinones and their derivatives play a critical role in a range of applications including catalysis, energy storage,^23,24^ and biological processes such as respiration and photosynthesis.^25−27^ In aprotic solvent, neutral quinones can undergo a one-electron reduction to a radical anionic semiquinone (Q^•–^). The semiquinone can undergo further one-electron reduction to an aromatic dianion (Q^2–^), as illustrated in Scheme 1.^26,28−30^ In aqueous or protic media, the same reduction is often coupled to a proton transfer, resulting in neutral semiquinone (QH^•^) or hydroquinone (QH_2_) species.^17,19,28^

Most computations apply a thermodynamic cycle to compute redox potentials.^17^ There are several important ingredients for such calculations to be accurate. Chief among them, the solvation model used must have a balanced description of the solvation of the oxidized and reduced species.^31^ Therefore, most benchmark studies have placed a heavy emphasis on testing solvation models for redox potential calculations.

Multiple redox benchmark studies have indicated that calculations are accurate to within a fraction of 1 V relative to experiments. Coote and co-workers^19^ studied 13 Q/Q^•–^ reduction potentials of para-quinones in a nonaqueous solvent, acetonitrile. They employed the composite G3(MP2)-RAD approach for the gas-phase calculations and Hartree–Fock (HF) or B3LYP solvation energy calculations with the conductor-like polarizable continuum model (C-PCM). They found that the HF and B3LYP solvation gave mean absolute deviations (MADs) of 70 and 120 mV compared to experimental redox potentials, respectively. They also tested the effect of using B3LYP for the gas-phase calculations, applying a constant correction factor of 280 mV derived by Guo and co-workers^20^ on the basis of ionization energy calculations, which they suggest would also be applicable to electron affinities. Guo and co-workers^20^ studied both gas-phase ionization potentials (IPs) and oxidation potentials of 270 diverse organic molecules and showed that using a density functional theory (DFT) with polarizable continuum model (PCM) gave an average error of 170 mV in acetonitrile after a constant shift of 280 mV applied to the IPs. Leszczynski and co-workers^32^ tested several different density functionals and solvation methods for calculating Q/Q^•–^ reduction potentials of several classes of molecules, including nine quinones, with MADs ranging from 30 to 300 mV. Grimme and co-workers compiled a set of 313 reduction and oxidation potentials (313ROP) for organic and organometallic compounds in various solvents and used the data to benchmark semiempirical quantum mechanical methods with implicit solvation.^33^

Several studies have also employed explicit solvation for computing reduction potentials of quinones. Zimmerman and co-workers^34^ found that DFT with implicit solvation generally worked well for Q/Q^•–^ reduction potentials, but in a few high-error outliers, using explicit (QM/MM) solvation reduced the errors significantly from 194 to 8 mV. They also found that implicit solvation gave a much larger MAD for Q^•–^/Q^2–^ reduction potentials under acidic aqueous conditions. These errors were reduced by including in the simulation a counterion that stabilizes the anionic species. Ghosh et al.^35^ and Tazhigulov et al.^36^ presented protocols for the accurate prediction of redox potentials using *ab initio* wave function methods with an effective fragment potential (EFP) to describe the solvent.^37^

More recently, several groups have applied machine learning (ML) approaches to model redox potentials. Liu and co-workers^38^ used ML to reduce errors from DFT calculations and tested it on Grimme’s ROP313 data set of organic and organometallic compounds. They found that with the best-performing ML corrections, errors were reduced by 23–49%, reducing the MAE from 430 to 220 mV. They also found that the ML approach is less sensitive to the DFT functional used.

The current study benchmarks theoretical calculations for 345 Q/Q^•–^ and 265 Q^•–^/Q^2–^ reduction potentials of quinones in an aprotic solvent. While it is desirable to test computational protocols against experimental data for a wide range of different molecules, as done, for instance, in the ROP313 set, here we take a different approach; we study a large data set of reduction potentials consisting of closely related organic molecules that have been measured using a common experimental setup and conditions. All of the experimental reference data used in this work were recently reported in a study by Prince, Dutton, and Gunner.^39^ The quinones range in size from 12 to 153 atoms and include several substituents including alkyl chains, halogens, polar protic and aprotic groups, and even charged groups such as sulfonates. The data set includes quinone derivatives such as adriamacyin and cerubidine, which are clinically approved drugs for treating cancer,^40−42^ natural dyes such as 2-hydroxy-1,4-naphthoquinone (henna),^43^ and synthetic dyes such as disperse red 9 and 1-(methylamino)-anthraquinone, which is used in bank security dye packs that get activated upon bank robberies.^44^

Having a large data set from a single source reduces the likelihood of random errors associated with different equipment and human error. The aprotic solvent conditions in which the experiments were carried out simplify calculations since they mitigate proton transfer with the solvent but also pose a challenge to some continuum solvation models that typically do not perform as well for low dielectric constants as for aqueous solvation.^19,45^ Furthermore, the focus on both sequential Q/Q^•–^ and Q^•–^/Q^2–^ potentials allows for a more stringent test of electronic structure methods and solvation models that must treat neutral, radical anionic, and dianionic solutes in a balanced way.

The aim of this study is to recognize sources of random and systematic errors in widely used DFT and implicit solvation models for sequential one-electron reduction potentials. Understanding the sources of these errors can help correct for them or develop improved computational methods and protocols.

## Computational Methods

Experimental Q/Q^•–^ and Q^•–^/Q^2–^ reduction potentials were reported recently by Prince et al. in dry N,N-dimethylformamide (DMF) solvent against a saturated calomel electrode (SCE).^39^ Most molecules are derivatives of three parent quinones: 1,4-benzoquinone (BQ), 1,4-naphthoquinone (NQ), and 9,10-anthraquinone (AQ). A few other quinone isomers or related aromatic nonquinones are also included (“others”). Specifically, Prince et al. reported experimental reduction potentials for 117 substituted BQs, 90 substituted NQs, 110 substituted AQs, and 33 miscellaneous quinones and nonquinones—350 molecules in total.^39^ In Supporting Information (SI) Table S1, we list those 350 molecules using indices 000 to 349 and include their chemical structures. Of those 350, we exclude 5 molecules from the analysis in this work: 3,x-dichloro-2-methoxycarbonyl-1,4-benzoquinone (116), where a structure could not be determined from the molecule name; doxorubicin (209) and cerubidine (210), which we identified as duplicates of adriamycin (192) and cerubidine (193); and 2-methoxy-3-amino-5-methyl-6-decaprenyl-1,4-benzoquinone (094) and reactive blue (164). The last two were excluded due to their high molecular weight, rendering the computations intractable. This resulted in a total of 345 Q/Q^•–^ reduction potentials. Experimental Q^•–^/Q^2–^ reduction potentials were not reported for 80 out of the 345 molecules,^39^ so we have 265 Q^•–^/Q^2–^ reduction potentials.

Quantum chemical calculations employed DFT with the Becke-3 Lee–Yang–Parr (B3LYP)^46,47^ functional and the 6-311++G(d,p)^48^ split-valence triple-ζ basis set. Unrestricted B3LYP was used for the open-shell one-electron reduced radicals. This combination of method and basis set is a popular choice that has been tested extensively for quinones in combination with implicit solvation.^28,49,50^ A mixed basis set was used for iodine-containing molecules, treating iodine with the def2TZVP basis set with an effective core potential^51^ and remaining atoms with 6-311++G(d,p). Frequency calculations were used to check for imaginary frequencies. If imaginary frequencies were found, then the structure was displaced along the scaled normal mode vector corresponding to that frequency and reoptimized until all positive frequencies were obtained.

Throughout this work, we refer to the initial (oxidized) quinone as the neutral quinone Q, the one-electron reduced species as the radical anion Q^•–^, and the fully reduced quinone as a dianion Q^2–^. However, several molecules included in this work have charged substituents such as sulfonates. In those cases, an additional negative charge is included in the computations, but we continue to use the Q/Q^•–^/Q^2–^ nomenclature for convenience, as these refer to the charge and oxidation state of the quinone backbone. Frontier orbitals for a few representative cases (two monosulfonate-AQs, one disulfonate-AQ, and one monosulfonate-NQ) indicate that the reduction in those molecules occurs on the quinone ring (as shown in SI Figure S1).

Since DMF is an aprotic solvent, we assume that all alkaline substituents such as amines (which are mostly anilines and therefore weak bases), remain deprotonated. Weak acids, such as alcohols, were kept protonated. However, sulfonates (R-SO_3_^–^) are kept deprotonated because they are strong acids.

Solvation energies in DMF were computed using four implicit solvation methods: a polarizable continuum model^52−54^ using the integral equation formalism (IEF-PCM),^53^ a conductor-like polarizable continuum model (C-PCM),^55,56^ a COnductor-like Screening MOdel (COSMO),^57^ and SM12.^58−60^ The C-PCM and COSMO implementations used in this work both employ the switching/Gaussian (SWIG) surface discretization method used to obtain smoother potential energy surfaces^61−63^ and therefore differ primarily by their treatment of outlying charge corrections and by their reaction field screening factor, *f*_ϵ_:
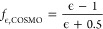
1
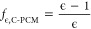
2

In the case of IEF-PCM, we tested the effect of using a single-point energy calculation on the gas-phase optimized geometries (IEF-PCM-cycle) as well as reoptimizing the molecule with PCM solvation (IEF-PCM-direct). On the other hand, C-PCM, COSMO, and SM12 calculations were carried out only as single-point energy calculations on the gas-phase optimized geometries (i.e., using the thermodynamic cycle).

Gas-phase and IEF-PCM calculations, including geometry optimizations, were computed with the Gaussian 16 software package.^64^ C-PCM, COSMO, and SM12 solvation energies were computed using the Q-chem 5.4 software package.^65^ SM12 calculations were parametrized based on CM5 partial atomic charges.^58,66^ For the C-PCM and COSMO calculations, a dielectric constant of 37.219 was used for DMF, obtained from the Minnesota Solvent Descriptor Database.^67^

In both Gaussian and Q-Chem, SCF convergence issues were encountered frequently for the doubly reduced states, especially in the gas phase where such states may be metastable. Those issues were resolved using SCF convergence options available in both software packages. Specifically, in Gaussian, we employed the YQC convergence option that performs linear searches and then switches to a default or quadratically convergent algorithm to complete the convergence.^68^ When that fails, SCF convergence is achieved with another method first and used as a guess for B3LYP. In Q-Chem, we used different SCF starting guess orbitals and/or changed the SCF algorithm until convergence. For SM12 calculations, the solvent self-consistent field procedure did not converge correctly for three anions, so the Merz–Singh–Kollman^69,70^ (for molecules 085 and 299) or CHELPG^71^ (for molecule 050) atomic charge model was used instead of CM5.

**Scheme 2:**
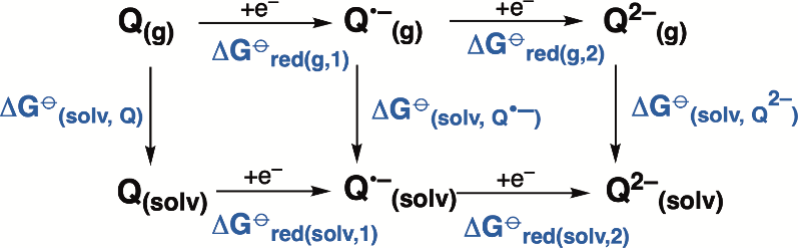
Born–Haber Thermodynamic Cycle^17^ for One-Electron Reduction From neutral to anionic semiquinone (reduction 1) and from anionic semiquinone to the two-electron reduced dianionic quinone (reduction 2). The upper leg shows gas-phase reduction free energies; the lower leg shows reduction free energies in solvent. Those two legs are connected by solvation free energy calculations (arrows pointing down).

From the thermodynamic cycle in Scheme 2, the free energy of reduction in solvent can be computed using:
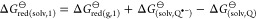
3
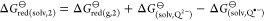
4

Δ*G*_red(solv,1)_^⊖^ and Δ*G*_red(solv,2)_^⊖^ represent the free energy due to reduction in solution of Q to Q^•–^ and Q^•–^ to Q^2–^, respectively. Δ*G*_red(g,1)_ is calculated by taking the difference of the sums of the electronic energies and thermal correction to Gibbs free energy of the reduced and oxidized forms.^19,34,72,73^ The thermal corrections are obtained from the frequency calculations of the gas-phase optimized structures. The reduction free energies are related to the absolute reduction potentials,
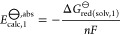
5
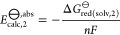
6where *n* represents the number electrons (here, 1) gained by the quinone from the electrode and *F* is Faraday’s constant, 23.061 kcal mol^–1^ V^–1^.^74^*E*_calc,1_^⊖abs^ and *E*_calc,2_^⊖abs^ represent the absolute theoretically predicted values of the standard potentials for reductions 1 and 2, respectively. We report all reduction potentials in units of mV.

The reduction potentials in eqs 5 and 6 are absolute potentials and therefore represent the Gibbs free energies required to bring a free electron from vacuum and add it to the molecule. On the other hand, the experimental reduction potentials were measured by Prince et al. under standard conditions relative to the SCE.^39^ To compare the computed reduction potentials to the experimental ones, it is necessary to subtract the absolute reduction potential of the SCE under the same conditions. Specifically, for reduction 1 and reduction 2,^75^
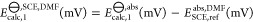
7
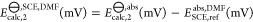
8

*E*_SCE,ref_^abs, DMF^ represents the SCE reduction potential, which is the free-energy change associated with the reference SCE anodic half-reaction, . The SCE potential is typically reported relative to the standard hydrogen electrode (SHE), . However, determining the absolute reduction potential of the SHE, and therefore SCE, is not trivial and has remained a topic of debate in the literature.^75−81^ Much of this debate revolves around the theoretical treatment of the proton hydration energy and free electron energy terms through work functions that account for surface effects. IUPAC recommended an SHE absolute potential close to 4.44 V.^82^ However, Kelly et al.^72,83^ and Isse et al.^75^ argue that a more suitable computational reference should use the standard hydration free energy of the proton instead of the real potential at the gas/liquid interface since the latter includes an energy contribution related to the surface potential at the interface between the two phases. They arrive at a reduction value of 4.28 V for the SHE. Recent computational studies have continued to indicate good agreement between computed and experimental redox potentials using an SHE reference of 4.44 V.^80,84^ Recently, Williams et al. obtained via nanocalorimetry techniques new values for the absolute SHE ranging from 4.11^85^ to 4.2 V^86,87^ while Shigeta et al. computed SHE’s potential to be 4.48^84^ and 4.52 V.^88^

In addition to the difficulty in knowing the absolute potential of the SHE reference, determining the relative potential of SCE to SHE for a nonaqueous solution is further complicated by liquid junction potentials arising from different ion mobilities in different solutions. Experimentally, such reduction potentials are sensitive to the solvent, electrolytes, and environmental factors, which may result in difficulties with reproducibility.^73,89,90^ Isse et al., using a reduction potential of 4.281 V for aqueous SHE, a +0.241 V potential for aqueous SCE vs SHE, and a 0.172 V liquid junction potential reported by Diggle and Parker for DMF solvent,^91^ arrive at an absolute reduction potential of 4.350 V for SCE with DMF.^75^

Often, this issue of absolute potentials can be avoided by using an internal reference such as the ferrocene/ferricenium redox couple.^73^ This led to multiple attempts to model the absolute reduction potential of ferrocene accurately.^92,93^ Prince et al.^39^ reported the reduction potential for ferrocene vs SCE as +524 mV using their experimental setup and conditions. Similarly, several studies reported computed hydrogen electrode (CHE) values as in refs (94−97). Since ferrocene and H_2_ have very different electronic and molecular structures than quinones, we did not use them in this study as an internal computational reference.

In light of the uncertainties surrounding the absolute reduction potential of SCE, we followed an approach similar to that of others^80,98^ and opted to report a direct correlation between the absolute theoretically calculated reduction potentials compared to the experimental values vs SCE. The computational values will therefore be offset from the experimental data by a constant reflected in the y-intercept. This constant, a fitted parameter, corresponds to the absolute reduction potential of the SCE if we assume no systematic errors. However, systematic errors that arise in our calculations will also be absorbed by this fitted y-intercept on top of the SCE offset. Since we cannot determine with certainty the magnitude of this systematic error, we thus cannot determine the exact SCE value. However, due to the large data set of 610 reduction potentials (345 Q/Q^•–^ and 265 Q^•–^/Q^2–^), we can compare subsets of this data to disentangle to some extent some sources of random errors (reflected by the MAE) and systematic errors (reflected by the y-intercepts).

The redox potential calculations were automated using the following algorithm:

1. Obtain SMILES string from molecule’s CAS number (if available) using CIRpy.
2. Convert the SMILES string into 3D molecular structures in Cartesian coordinates using RDKit.^99^
3. For molecules without CAS numbers, generate initial coordinates using IQmol.
4. Initialize submitting parallel gas-phase optimization jobs by preparing necessary input files for each of the neutral, radical, and anionic state of every molecule.
5. Check for output file errors. In case of SCF convergence errors, resubmit with a different SCF convergence algorithm.
6. Check for negative frequency occurrences. If found, resubmit optimization from last geometry displaced along scaled normal modes.
7. Extract gas-phase optimized coordinates.
8. Run single-point solvation energy calculations.
9. Extract the gas-phase and solvent electronic energies and thermal correction to the Gibbs free energy and use them to calculate reduction potentials for Q/Q^•–^ and Q^•–^/Q^2–^ .

### Statistical Analysis

Experimental and computed reduction potentials for the different solvation models (IEF-PCM-cycle, IEF-PCM-direct, C-PCM, COSMO, and SM12) and for each of the two reduction reactions (Q/Q^•–^ and Q^•–^/Q^2–^) were plotted and fit using a linear regression with the slope constrained to 1. As discussed in the Computational Methods section, computed redox potentials are absolute potentials, while the experimental redox potentials were measured relative to SCE.

The computed and experimental data have different standard deviations: σ_exp_ = 334 mV while σ_comp_ varies from 390 to 401 mV for different solvation models in the case of Q/Q^•–^, and σ_exp_ = 254 mV while σ_comp_ varies from 284 to 299 mV for different solvation models for Q^•–^/Q^2–^. The wider distribution of computed potentials can be attributed to systematic errors and outliers in the computed data.

The BQ, NQ, AQ, and other redox potentials were all fit together using a general linear regression for all molecules. Then, the four parent groups (BQ, NQ, AQ, and other) were fit separately. If the Q/Q^•–^ and Q^•–^/Q^2–^ calculations and experiments for the four groups (BQ, NQ, AQ, and other) are of similar quality, then it is expected that they should have similar y-intercepts and MAEs. Therefore, fitting the data both ways (together and individually) helps delineate possible sources of random and systematic error in the different groups.

Among the arsenal of statistical tools available, we chose to quantify the discrepancy between computed and experimental values using two metrics: the Pearson correlation coefficient,
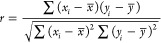
9and the mean absolute error (MAE, in mV),
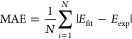
10

The first metric, *r*, represents the linearity of the correlation between computed and experimental potentials. The second metric, the MAE, represents the average deviation of the data from the idealized (linear) fit. This is not a deviation from the experimental data, which is not used here since those are offset by the SCE absolute potential. Those two metrics were chosen, along with the choice to constrain the slope to 1, because they are invariable to the choice of reference axis (experimental vs computed). Other widely used metrics, such as the coefficient of determination (*R*^2^), depend on a null hypothesis that is sensitive to the standard deviation of the data.

All metrics were calculated using Scikit-learn library version 1.2.2 using the sklearn.metrics^100^ module or numpy version 1.25.2.

## Results and Discussion

### Q/Q^•–^ Reduction Potentials

**Figure 1:**
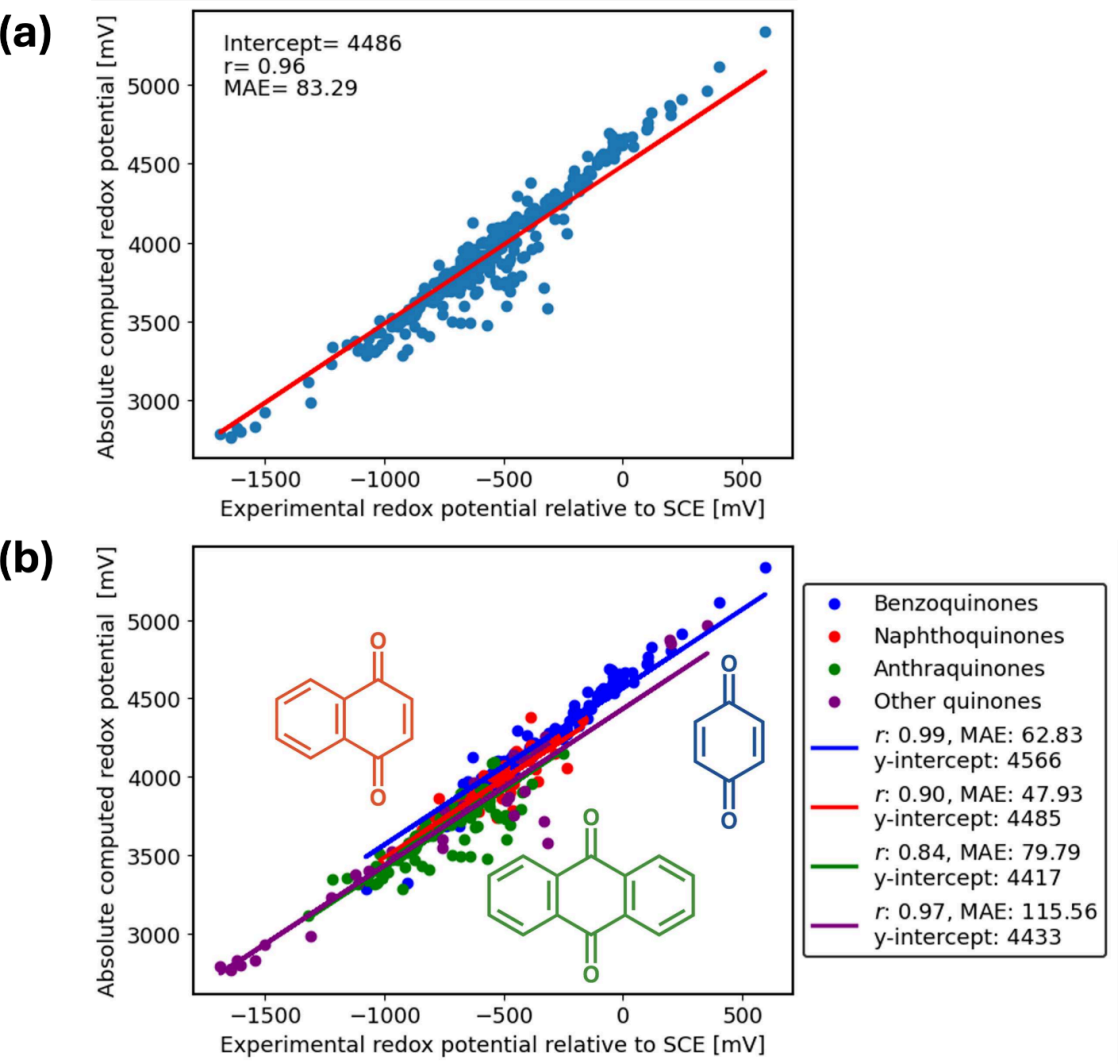
(a) Correlation between Q/Q^•–^ experimental reduction potentials relative to SCE and absolute calculated reduction potentials for *N* = 345 total studied quinone derivatives in DMF using the IEF-PCM-cycle approach. (b) Correlation between experimental reduction potentials and absolute calculated ones fitted separately for 4 groups: 115 benzoquinones (blue), 90 naphthoquinones (red), 107 anthraquinones (green), and 33 others (purple). Experimental data were obtained from Prince et al.^39^

The correlation between experimental Q/Q^•–^ reduction potentials relative to the SCE and the absolute computed reduction potentials is illustrated in Figure 1. In this figure, we constrained the slope to 1.00. The linear fit gives an *r* value of 0.96, an MAE of 83.29 mV, and a y-intercept of 4486 mV, as shown in Figure 1(a). If we assume that the SCE reduction potential is 0.2412 V above SHE in aqueous media^90,101^ and that there is a liquid junction potential of 0.172 V for DMF with tetraalkylammonium salts,^91^ then this means that SCE in DMF has a reduction potential of 0.069 V relative to SHE. A value of 4.486 V for SCE translates to 4.417 V for SHE. The standard deviation associated with this value is 0.092 V (based on the standard deviation of data relative to the fit). 4.417 V lies between the commonly used values of 4.28 and 4.44 V.^72,75,82,83^ However, any systematic errors in the gas-phase electron affinity calculations and in the solvation energies are reflected in this value.

A linear regression analysis without any constraint instead yields a slope of 1.1491 and a lower MAE of 69.76, as shown in Figure S2(a). The large slope is unphysical and indicative of a systematic error in the calculations, reflecting a larger variance in the computed reduction potentials compared to the experimental ones.

To better understand the source of this error, we plot in Figure 1(b) the linear regression analysis for BQ, NQ, AQ, and other quinones separately. We notice two trends. First, the different groups have different average redox potentials, with BQ on average having higher (more positive) reduction potentials than NQ and with AQ having the lowest potentials. Second, the y-intercepts follow the same trend if the slope is constrained to 1: BQ > NQ > AQ. This is indicative of a systematic error that is different for each group. Comparing Figure S2(a) and Figure 1(b), we can now attribute the larger-than-1 slope in Figure S2(a) to a systematic error related to the length of the conjugation or the ring size of the quinone. This error is not related to the total size of the system (i.e., including substituents); when accounting for substituents, the average sizes of BQs, NQs, AQs, and other quinones are comparable, with average sizes of 29, 30, 35, and 27 atoms, respectively. Moreover, a plot of individual absolute errors (relative to the fit) vs number of atoms yields a plot with no linear correlation (*R*^2^ = 0.002 for linear regression).

In Figure S2(b), we include a linear regression analysis for BQ, NQ, AQ, and other quinones separately but without constraining the slope to 1. We find that the slope now varies considerably for the different subgroups (1.195 for BQ, 0.964 for NQ, 0.859 for AQ, and 1.041 for others). Since the slope is not always larger than 1 within each group, this again indicates that the overall slope, 1.1491 in Figure S2(a), is related to the ring size and is not a constant overestimation for all systems. This error may originate from the gas-phase electron affinity calculations; DFT has been shown to exhibit size-dependent errors for ionization potentials.^102,103^

Next, we report on the random error. From Figure 1(b), NQs exhibit a lower MAE (47.93 mV) compared to that of BQs (62.83 mV), which, in turn, is lower than that of AQs (79.79 mV). However, when the slope is not constrained to 1 (Figure S2(b)), the trend in MAEs increases with the ring size (43.34 mV for BQ, 48.54 mV for NQ, 77.35 mV for AQ, and 113.56 mV for others). This change in trend is tied to the different slopes of the subgroups; for example, NQ has a slope closest to unity in Figure S2(b), so the MAE remains small when the slope is constrained to 1.

There are several factors that could contribute to the different intrinsic slopes and MAEs of the different groups. One of those factors is the nature of the substituents. For example, protic substituents which are not properly captured by implicit solvation models may be involved in hydrogen-bonding interactions or even acid/base chemistry.^34^ While the measurements by Prince et al. were carried out in aprotic solvent and care was taken to minimize the amount of moisture,^39^ even a few millimoles of water can alter the apparent reduction potential of quinones in DMF.^104^ Therefore, such systems may give larger errors that are more pronounced for protic quinones. This is supported by the data in Table 1, where titratable quinones give, on average, larger MAEs than nontitratable quinones. In the table, we categorized quinones as titratable if they have a group that may act as an acid or base, such as amines, alcohols, and sulfonates, and as nontitratable if they have only groups such as halogens, nitro, cyano, and acetoxy. The “other” quinones are the only group that does not follow the MAE(titratable) > MAE(nontitratable) trend because it constitutes a small sample size with several bulky outliers that skew the errors. Another trend emerges in Table 1: The number of titratable and number of nontitratable residues are not equally divided among the parent quinones, which introduces a bias: BQ has mostly nontitratable substituents, NQ has an equal number of titratable and nontitratable substituents, and AQs have mostly titratable residues. Ideally, the three groups should be tested having similar substituent types. However, likely due to factors such as ease of synthesis, stability, and solubility, the natures of the substituents on BQ, NQ, and AQ are not the same. This serves as a possible explanation for why the MAEs increase across the series BQ < NQ < AQ (when the slope is not constrained to 1, see Figure S2(b)).

**Table 1:** MAEs for Q/Q^•–^ Reduction Potentials for Molecules with Titratable versus Nontitratable Subsituents Using the IEF-PCM-Cycle Approach^a^

	MAEs for all	MAEs for BQs	MAEs for NQs	MAEs for AQs	MAEs for others
Titratable	83.9 (*N* = 140)	107.2 (*N* = 16)	57.6 (*N* = 45)	93.7 (*N* = 68)	96.6 (*N* = 11)
Nontitratable	55.7 (*N* = 205)	55.9 (*N* = 99)	41.3 (*N* = 45)	55.6 (*N* = 39)	125.0 (*N* = 22)

a *N* represents the total number of titratable or nontitratable molecules across all quinones or across every parent subgroup.

In SI Table 2, we further break down the errors associated with different substituents. We find in particular that cyano, sulfonates, acetoxy, amine, and hydroxy groups have higher MAEs than the computational average for all quinones, whereas halogens, nitro, oxy, and thio substituents give MAEs below the average. Such errors may not always be systematic; several anecdotal examples suggest that errors are not easy to predict from structure. The molecules that give the largest errors such as 333 and 334 (absolute errors relative to fit of 591 and 441 mV, respectively) have pyramidalized aromatic rings due to bulky isoproyl and tetrabutyl substituents adjacent to each other, inducing a large steric strain on the quinone ring. Anthraquinones with several hydroxy groups such as 203 and 207 (absolute errors relative to fit of 443 and 316 mV, respectively) also tend to give large errors due to intramolecular hydrogen bonding and proton rearrangements upon reduction. Another molecule, 1-amino-2-sulfonate-9,10-anthraquinone (ID 157), has a large error relative to fit of 399 mV. Introducing a bromo group para to the amine in 1-amino-4-bromo-2-sulfonate-9,10-anthraquinone (ID 163) reduces the error considerably to 4 mV. The orbital involved in this reduction is similar for the two molecules and involves the quinone ring (as shown in Figure S1). While one potential explanation is that the bromo group reduces the basicity of the amino group through its electron-withdrawing character, this example, along with the previous two, demonstrates the difficulty in trying to correlate the source of error for reduction potentials to structural elements such as substituents.

**Figure 2:**
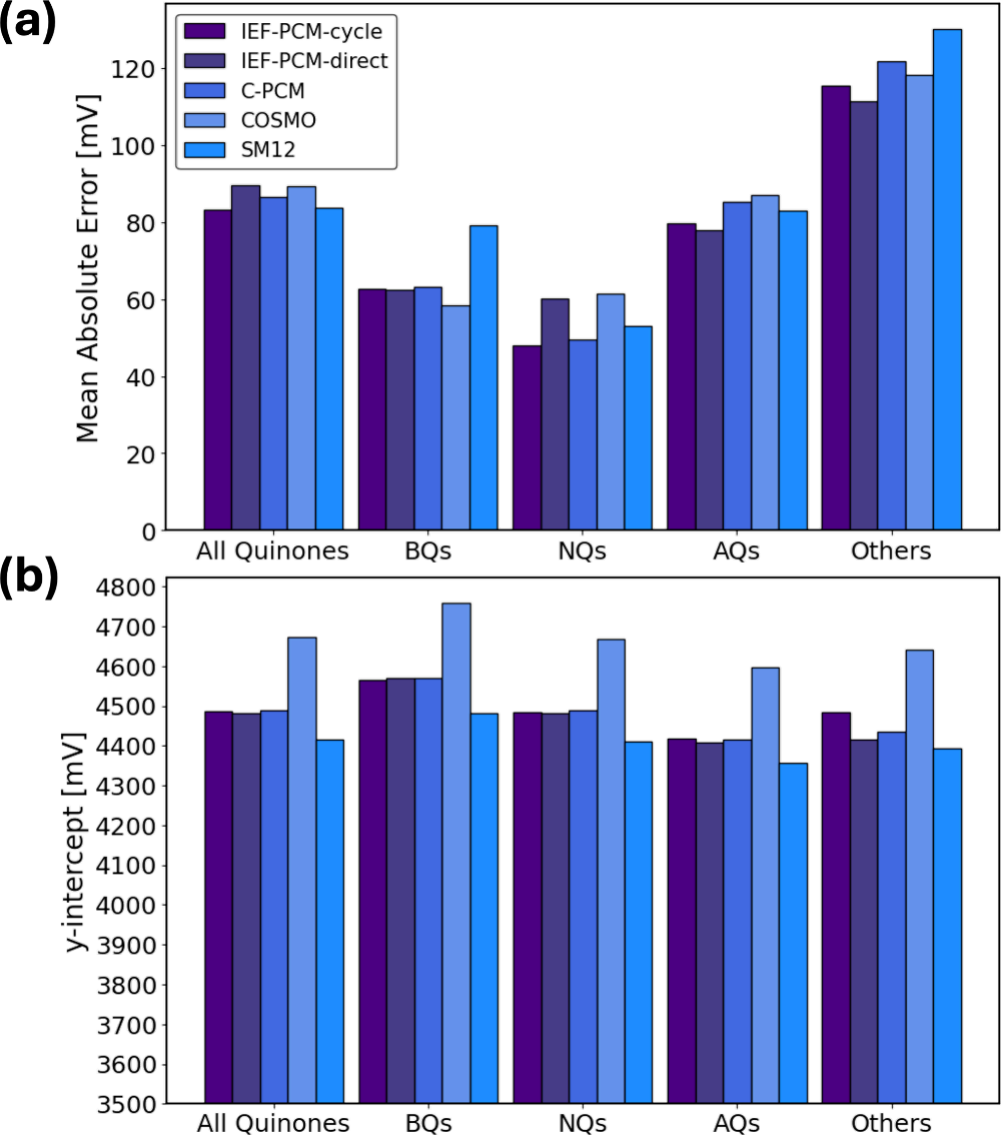
(a) Mean absolute errors (MAEs) for the linear regression between computed and experimental Q/Q^•–^ reductions for IEF-PCM-cycle, IEF-PCM-direct, C-PCM, COSMO, and SM12 considering all quinones or fitting them separately into groups: BQ, NQ, AQ, and others. (b) y-intercepts for the linear regression between computed and experimental Q/Q^•–^ reductions for IEF-PCM-cycle, IEF-PCM-direct, C-PCM, COSMO, and SM12 considering all quinones or fitting them separately into groups: BQ, NQ, AQ and others.

Next, we test the effect of carrying out the optimization and frequency calculations directly in the continuum solvent reaction field. This approach, referred to as IEF-PCM-direct instead of IEF-PCM-cycle, does not appreciably change the results of the calculation for Q/Q^•–^ reductions (Figure S3). We also test three other solvation models: C-PCM, COSMO, and SM12. The linear regressions, analogous to Figure 1, are shown in Figures S4–S6 in the SI. In Figure 2, we summarize the results of the linear regressions by comparing the MAEs and y-intercept values fitted by the different models. Generally, the MAEs for the different solvent models are comparable. However, COSMO solvation results in y-intercept values that are 180–280 mV higher than for the other models. This is consistent with a recent benchmark study by Tomanik et al.^80^ which indicates an optimal fitted value for the SHE absolute potential that is between 160 to 320 mV higher for COSMO than for IEF-PCM for different data sets. The y-intercept for C-PCM (4488 mV for all quinones) is closer to that of the IEF-PCM model (4486 mV) than COSMO (4673 mV).

### Q^•–^/Q^2–^ Reduction Potentials

**Figure 3:**
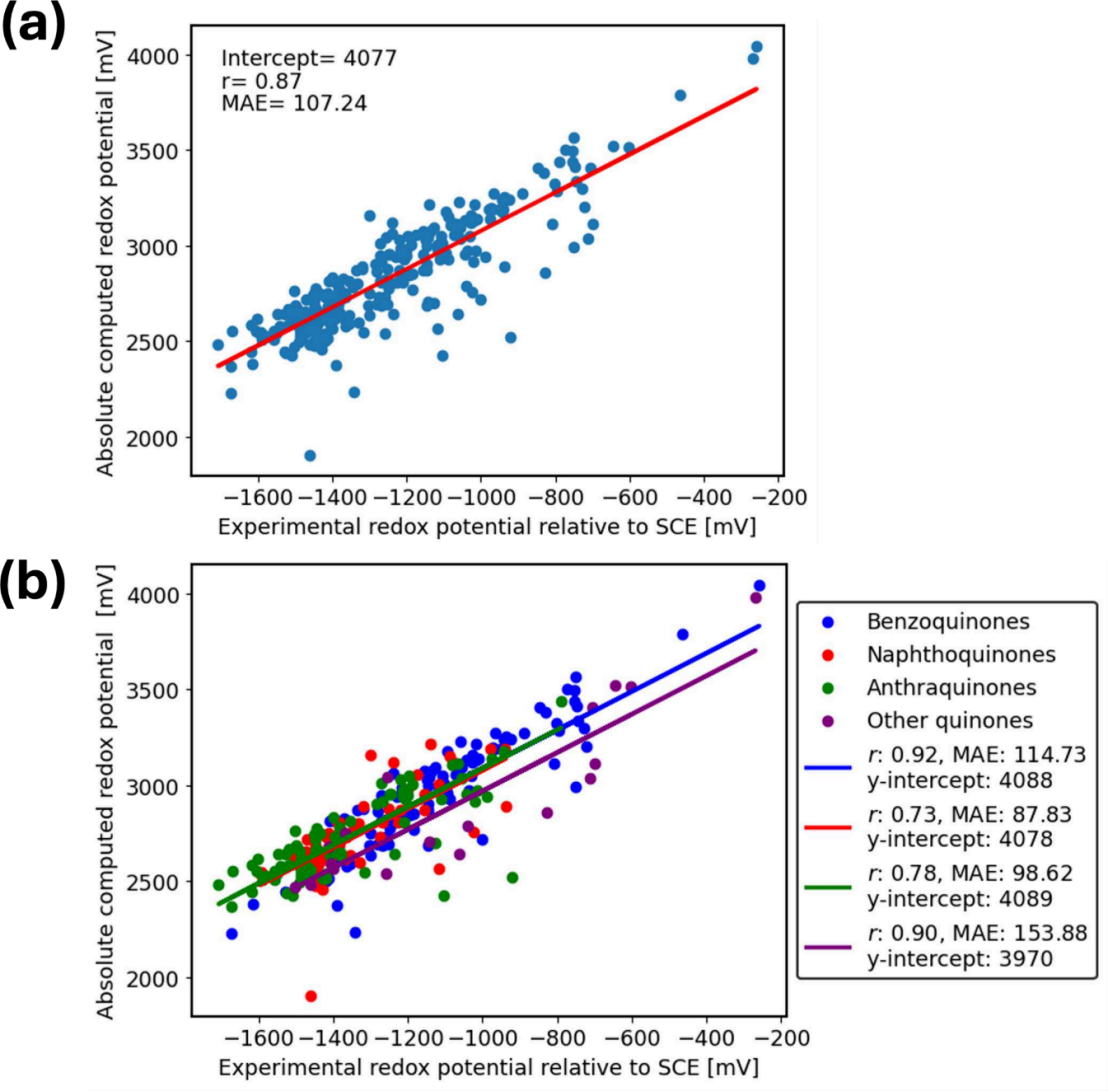
(a) Correlation between Q^•–^/Q^2–^ experimental reduction potentials relative to SCE and absolute calculated reduction potentials for *N* = 265 total studied quinone derivatives in DMF using the IEF-PCM-cycle approach. (b) Correlation between experimental reduction potentials and absolute calculated ones fitted separately for 4 groups: 89 benzoquinones (blue), 75 naphthoquinones (red), 83 anthraquinones (green), and 18 others (purple). Experimental data were obtained from Prince et al.^39^

The correlation between experimental Q^•–^/Q^2–^ reduction potentials relative to the SCE and the absolute computed reduction potentials is shown in Figure 3. Compared to the first reduction Q/Q^•–^, there are several notable differences in the quality of the computations for the second reduction potentials Q^•–^/Q^2–^. First, the Q^•–^/Q^2–^ linear regression y-intercepts are reduced by almost 400 mV compared to those for Q/Q^•–^. This is indicative of a large difference in the systematic errors of the two data sets. Second, the Pearson correlation coefficient *r* is smaller for the Q^•–^/Q^2–^ data compared to those of Q/Q^•–^. This indicates a larger random error for the second reduction compared to the first reduction. Those random and systematic errors are explored further through IEF-PCM-direct and by using other solvation models.

**Figure 4:**
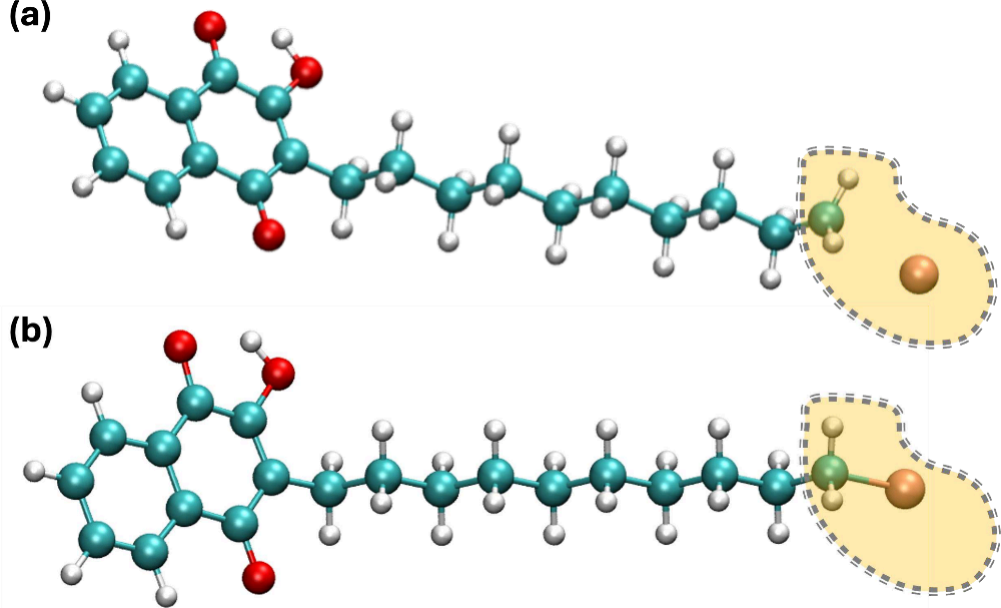
(a) Structure of the 2-hydroxy-3-(10-bromodecyl)-1,4-naphthoquinone dianion (molecule ID 295) optimized in the gas phase. This structure was used for single-point solvation energy calculations for IEF-PCM-cycle, C-PCM, COSMO, and SM12. (b) The same system optimized in the continuum solvent reaction field and used in IEF-PCM-direct.

One likely source of random error in the case of the second reduction is the lower stability of both species involved in the reduction. Q^•–^ is a radical anion, which is already negatively charged. Further reduction leads to a doubly charged dianion Q^2–^. Those species do not always remain stable in DMF, as reflected by the fact that reduction potentials could not be measured for all 350 molecules in the Prince et al. cyclic voltammetry experiments.^39^ When using the thermodynamic cycle, the Q^•–^ and Q^2–^ electronic structures and geometries are optimized in the gas phase where, in the absence of stabilizing interactions of a solvent or counterion, they may be metastable with frontier orbitals embedded in a continuum of other nearby virtual states.^105^ This is also manifested in SCF convergence issues, particularly for the dianions, which had to be resolved using varying SCF convergence algorithms. This led to some clear outliers in the data. One example, molecule 295 (2-hydroxy-3-(10-bromodecyl)-1,4-naphthoquinone), has an absolute error of 711 mV in IEF-PCM-cycle compared to the experiment. The source of the error is that the SCF algorithm converged to a wave function where the reducing electron was added to the Br orbital instead of the aromatic quinone ring, resulting in an optimized structure where the bromine group detaches from the rest of the molecule (Figure 4).

**Figure 5:**
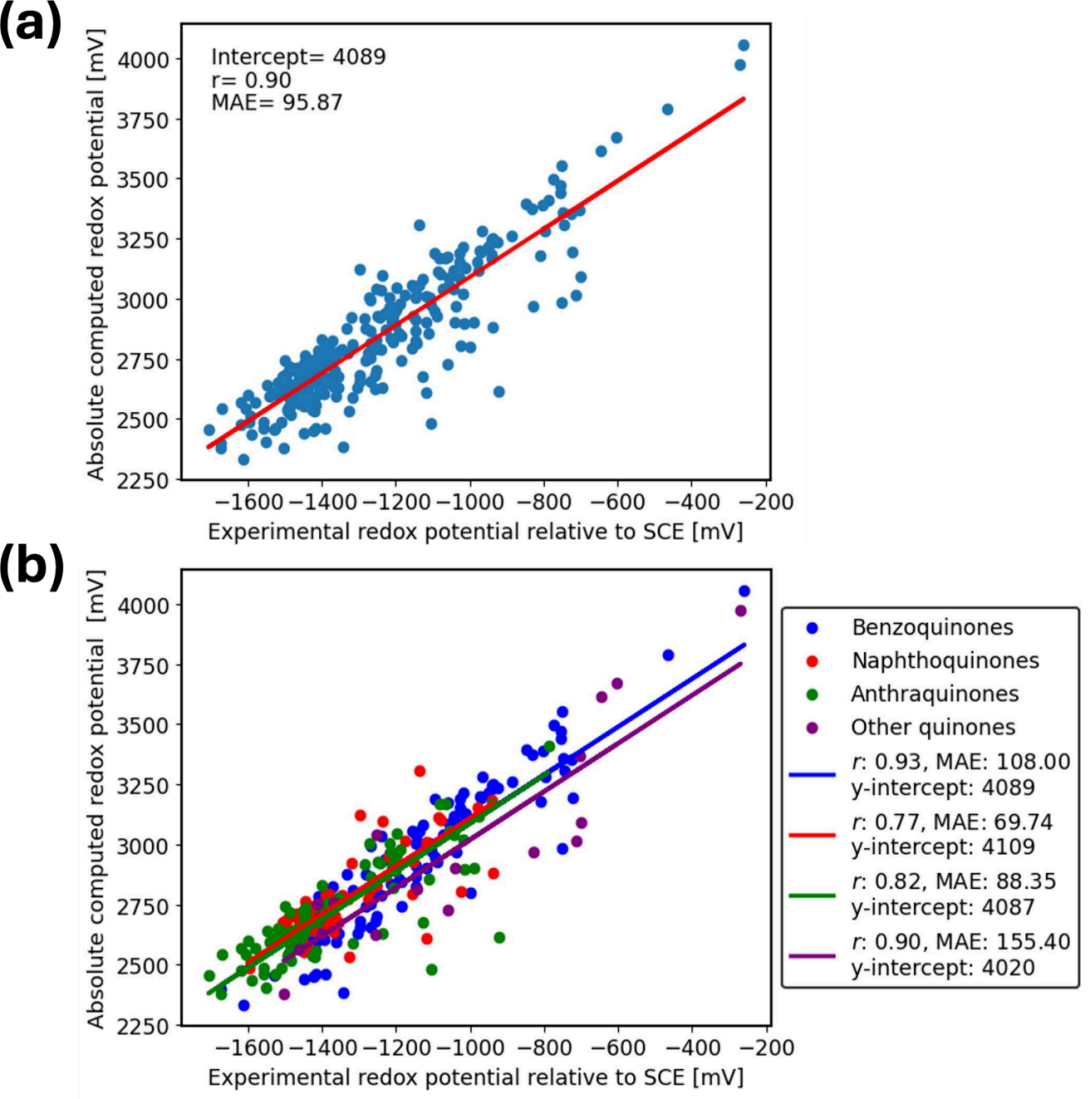
(a) Correlation between Q^•–^/Q^2–^ experimental reduction potentials relative to SCE and absolute calculated reduction potentials for *N* = 265 total studied quinone derivatives in DMF using the IEF-PCM-direct approach. (b) Correlation between experimental reduction potentials and absolute calculated ones fitted separately for 4 groups: 89 benzoquinones (blue), 75 naphthoquinones (red), 83 anthraquinones (green), and 18 others (purple). Experimental data were obtained from Prince et al.^39^

To mitigate errors stemming from SCF convergence in IEF-PCM-cycle, we test the IEF-PCM-direct approach, where the SCF algorithm and geometry optimizations are carried out under the stabilizing effect of the solvent reaction field instead of the gas phase^106^ (Figure 5). This led to improved SCF convergence in most cases, as well as a small improvement in the *r* value for the overall fit. For some specific molecules, the reduction in error was dramatic. With the IEF-PCM-direct approach, the absolute error for molecule 295 is 42 mV, compared to the 711 mV error using IEF-PCM-cycle (Figure 4). This is consistent with studies showing that optimizing molecules directly in solvent usually has only a limited effect unless there are significant structural changes induced by the solvation.^107^ Here, the structural changes stem from the difference in the SCF convergence in the gas phase compared to the IEF-PCM.

The linear regression for Q^•–^/Q^2–^ reduction potentials using IEF-PCM-cycle without setting the slope to 1 is shown in SI Figure S7. The slopes for the individual subgroups decrease in the order BQ > NQ > AQ, exactly as in the case for Q/Q^•–^ (Figure S2). Again, this error may be due to the unequal distribution of titratable and nontitratable residues across the three subgroups.

**Figure 6:**
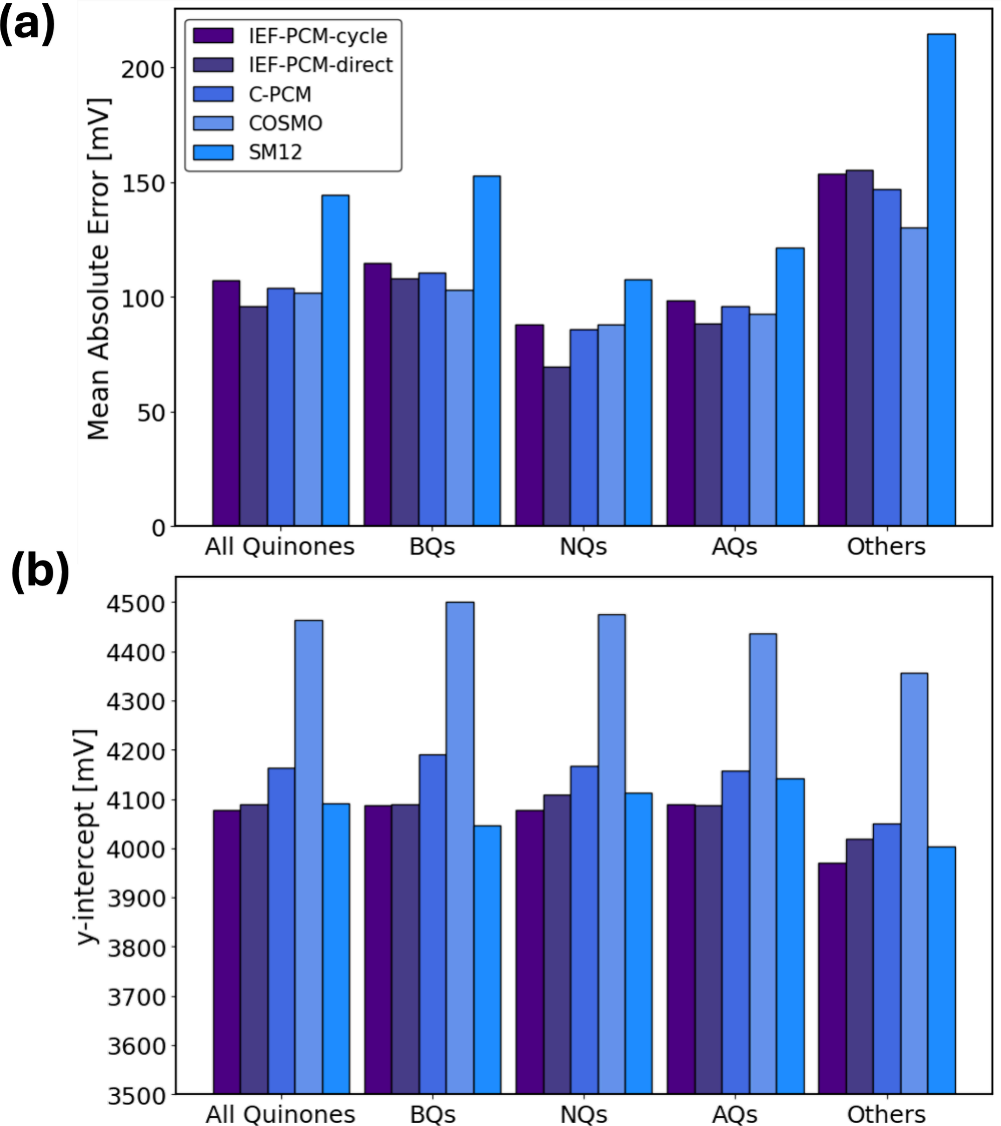
(a) Mean absolute error (MAE) for the linear regression between computed and experimental Q^•–^/Q^2–^ reduction potentials for IEF-PCM-cycle, IEF-PCM-direct, C-PCM, COSMO, and SM12 considering all quinones or fitting them separately into groups: BQ, NQ, AQ, and others. (b) y-intercept for the linear regression between computed and experimental Q^•–^/Q^2–^ reduction potentials for IEF-PCM-cycle, IEF-PCM-direct, C-PCM, COSMO, and SM12 considering all quinones or fitting them separately into groups: BQ, NQ, AQ, and others.

In Figure 6, we summarize the results of the linear regression analyses for IEF-PCM-cycle, IEF-PCM-direct, C-PCM, COSMO, and SM12 for Q^•–^/Q^2–^ by comparing their MAEs and y-intercepts. The linear regressions, analogous to Figure 3, are shown in Figures S8–S10 in the SI.

Generally, MAEs for IEF-PCM-direct are smaller than those for IEF-PCM-cycle because carrying out the optimization in PCM mitigates issues with SCF convergence affecting several molecules. Here, it would be more appropriate to compare C-PCM, COSMO, and SM12 data with the IEF-PCM-cycle data, since in all cases the same gas-phase structures were used. For instance, molecule 295 consistently introduces errors across the three solvation models, causing an error relative to the fit of 943 mV in SM12, 791 mV in COSMO, 753 mV in C-PCM, and 711 mV in IEF-PCM. When comparing the four solvent models, C-PCM and COSMO give slightly lower or comparable MAEs relative to IEF-PCM-cycle. On the other hand, SM12 calculations have a higher MAE across all subgroups. This is likely due to the CM5 charge model which is based on a Hirshfeld population analysis that works well for neutral and cationic species but can be problematic for anions.^108^ This error was not as pronounced in SM12 for the Q/Q^•–^ reduction, where it generally performed similar to if not better than other solvation models.

In both the Q/Q^•–^ data (Figure 2) and the Q^•–^/Q^2–^ data (Figure 6), we find that the other molecules (which includes a few quinones and nonquinones) exhibit the largest MAEs across all solvation models. These “other” molecules have the smallest sample size (*N* = 33 for Q/Q^•–^ and *N* = 18 for Q^•–^/Q^2–^) and include several sterically hindered outliers that increase their MAEs for both redox potentials. Focusing on BQs, NQs, and AQs instead, we find a shift in the error trends when we compare their Q^•–^/Q^2–^ MAEs to those of Q/Q^•–^. For the second reduction, Q^•–^/Q^2–^, BQ exhibits the highest MAE across all solvation models. This is likely because its negative charge (−1 for Q^•–^ and −2 for Q^2–^) is more concentrated on a smaller ring, posing a challenge to implicit solvation models that perform better when solvating more delocalized charges.^58^ In general, multiply charged anions may cause difficulties for both density functional theory, due to self-interaction errors, and solvent continuum models.^109^

Figure 6(b) shows the y-intercept values for the different solvation models. IEF-PCM-cycle (overall y-intercept = 4077 mV), IEF-PCM-direct (4089 mV), and SM12 (4092 mV) all give similar y-intercepts that are within 15 mV of each other. C-PCM has a y-intercept (4164 mV) that is higher across all subgroups. However, COSMO again gives a significantly higher intercept value, 4469 mV. While this was also true for the Q/Q^•–^ data (Figure 2), there the difference between COSMO and IEF-PCM-cycle was 187 mV. Here, the difference is more pronounced, with COSMO giving a y-intercept that is 387 mV above IEF-PCM-cycle and 305 mV above C-PCM.

In all solvation models, the y-intercept calculated for the Q^•–^/Q^2–^ reduction is smaller than that of Q/Q^•–^. Therefore, systematic errors between Q/Q^•–^ and Q^•–^/Q^2–^ reductions exist for all solvation models. However, this systematic error appears smallest for COSMO, which gives a Q^•–^/Q^2–^ y-intercept of 4464 mV compared to a Q/Q^•–^ y-intercept of 4673 mV, a difference of 209 mV. For the other solvation models (IEF-PCM, C-PCM, and SM12), this difference is closer to 320–410 mV.

## Conclusions

This benchmark of 610 reduction potentials from 345 quinones indicates several sources of systematic and random errors. The most prominent systematic error comes from the difference between the first reduction potential (Q/Q^•–^) and the second reduction potential (Q^•–^/Q^2–^). The latter potentials are lower than the first by around 320–410 mV with the IEF-PCM, C-PCM, and SM12 models. COSMO shrinks this difference to around 210 mV and therefore appears to have a more balanced description of solvation energies of neutral, anionic, and dianionic molecules compared to the other solvation models. Part of this systematic difference between Q/Q^•–^ and Q^•–^/Q^2–^ potentials may arise from self-interaction errors in density functional theory, which is larger for molecules with a higher negative charge.

Generally, MAEs (indicative of random errors) for Q^•–^/Q^2–^ reduction potentials are also higher than those for Q/Q^•–^. Within each group, we find a few sources of systematic error. For Q/Q^•–^, the ring size introduces an error which may be attributed to a lack of size extensivity of electron affinities computed with density functional theory. A second source of error is the nature of the substituents, with nontitratable substituents giving lower energies on average than titratable ones. Titratable substituents may undergo protonation or deprotonation events that alter the apparent reduction potential experimentally.

In Q^•–^/Q^2–^, the instability of the dianionic species, sometimes manifesting in SCF convergence issues, is another source of random error. This is partially mitigated by carrying out the SCF convergence in the PCM solvation field. The solvation of molecules with a higher charge concentration, as seen, for instance, in BQ Q^2–^ species where the −2 charge is localized on a smaller ring, contributes to larger MAEs as well.

While this study still indicates the usefulness of DFT calculations in predicting reduction potentials of quinones, there is still work needed to recognize patterns associated with computed reduction potentials and their agreement with experiments. The protocol used to run the calculations has been automated and made available on GitHub for further testing of different wave function methods, density functionals, basis sets, and/or solvation models. While other recent benchmark studies have focused on using a more comprehensive benchmark set that includes a wide range of molecules, we emphasize that there is also value in using a large database of closely related molecules (here, 345 quinones) measured under similar conditions to better resolve the sources of random and systematic errors.

## Data Availability

The calculation of 345 Q/Q^•–^ and 265 Q^•–^/Q^2–^ reduction potentials using different solvation methods requires thousands of quantum chemical calculations and their analysis. Therefore, the workflow and data analysis were largely automated through a series of Python and Bash scripts made available in a GitHub repository, https://github.com/gozem-gsu/Redox-Potential-Protocol. The repository also includes the B3LYP/6-311++G(d,p) optimized geometries of the 345 molecules in different redox states and all the data generated in this work. These scripts can be readily adopted to test different methods, basis sets, and solvation models or different molecules and properties.

## Supporting Information Available

Linear regression analysis of Q/Q^•–^ and Q^•–^/Q^2–^ absolute reduction potentials against experimentally determined reduction potentials relative to SCE for IEF-PCM-cycle (without constraining the slope to 1), IEF-PCM-direct, C-PCM, COSMO, and SM12; table of all molecular structures included in this benchmark and MAEs for Q/Q^•–^ reduction potentials for molecules with different substituent types (PDF)
Spreadsheet files listing experimental and computed (with IEF-PCM-cycle, IEF-PCM-direct, C-PCM, COSMO, and SM12) reduction potentials for all molecules, with categories for substituent type (ZIP)
