# Supplementary material for: First and Second Reductions in an Aprotic Solvent: Comparing Computational and Experimental One-Electron Reduction Potentials for 345 Quinones
Source: J Chem Theory Comput. 2024 Jul 6;20(14):6227–40. doi: 10.1021/acs.jctc.4c00602 (PMC11270834; doi:10.1021/acs.jctc.4c00602)
Supplement: Supplementary file 1 — ct4c00602_si_001.pdf [file ct4c00602_si_001.pdf]

Supporting Information for: First and Second Reductions in an Aprotic Solvent: Comparing Computational and Experimental One-electron Reduction Potentials for 345 Quinones

Sarah Elhajj[†], Samer Gozem[⋆,†]

Email: sgozem@gsu.edu
[†]Department of Chemistry, Georgia State University, Atlanta, Georgia, 30302, United States

Molecule ID	Structure	Size	Molecule ID	Structure	Size
000	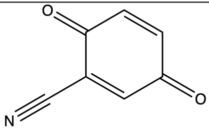	13	008	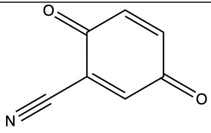	12
001	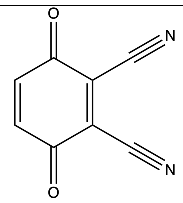	14	009	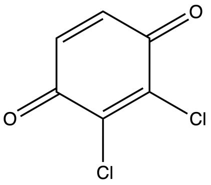	12
002	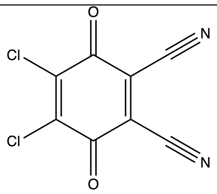	14	010	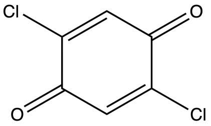	12
003	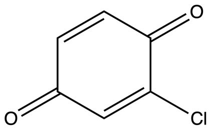	12	011	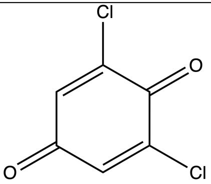	12
004	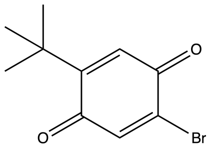	24	012	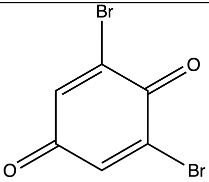	12
005	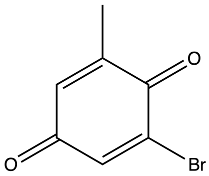	15	013	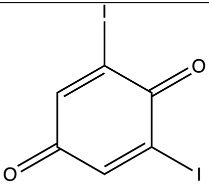	12
006	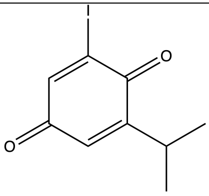	21	014	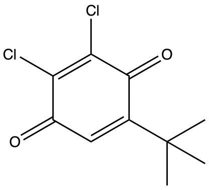	24
007	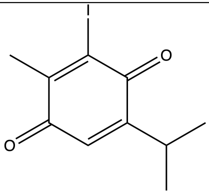	24	015	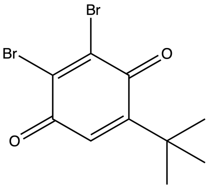	24

**Molecule ID**	**Structure**	**Size**	**Molecule ID**	**Structure**	**Size**
016	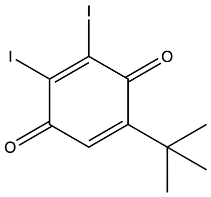	24	023	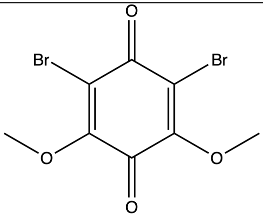	20
017	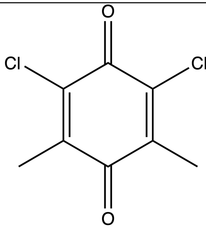	18	024	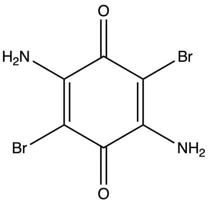	16
018	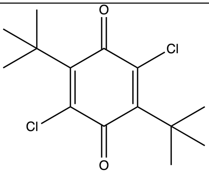	36	025	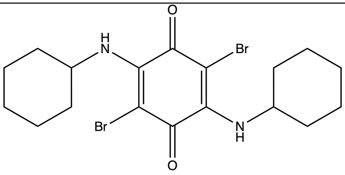	48
019	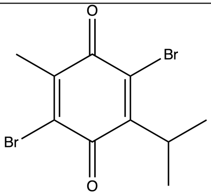	24	026	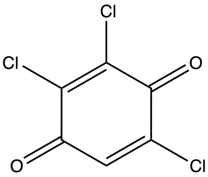	12
020	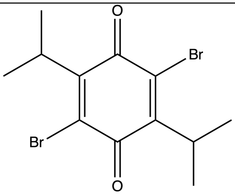	30	027	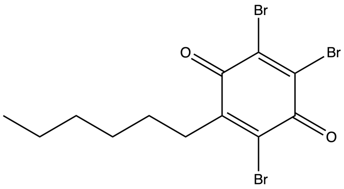	30
021	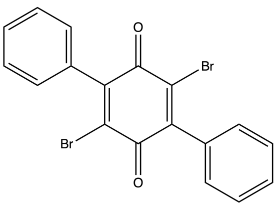	32	028	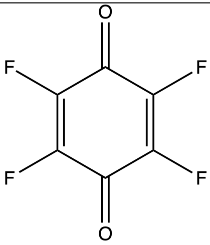	12
022	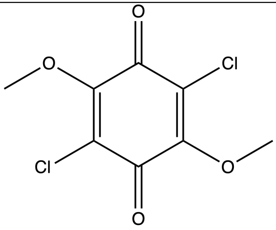	20	029	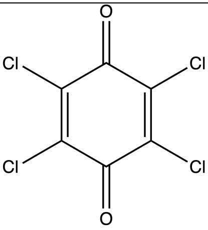	12

Molecule ID	Structure	Size	Molecule ID	Structure	Size
030	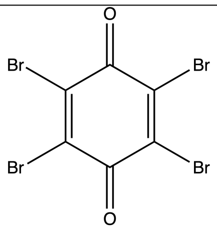	12	037	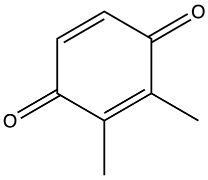	18
031	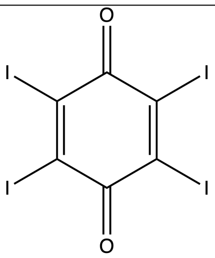	12	038	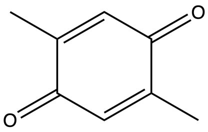	18
032	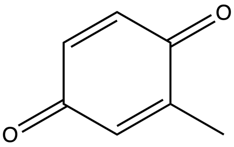	15	039	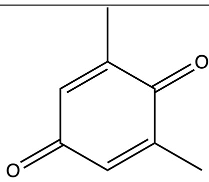	18
033	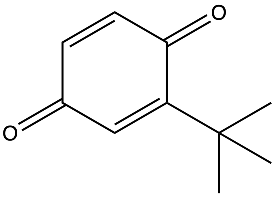	24	040	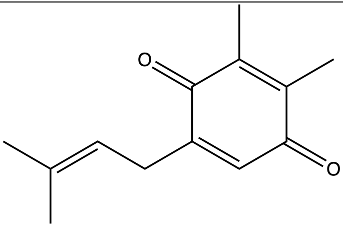	31
034	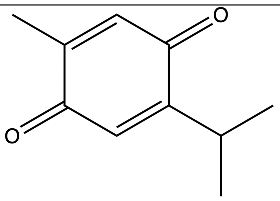	24	041	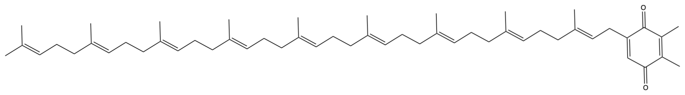	135
035	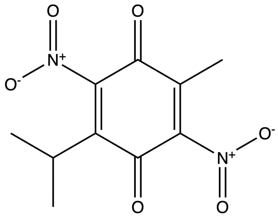	28	042	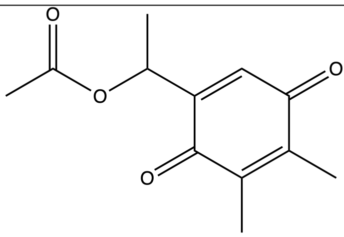	30
036	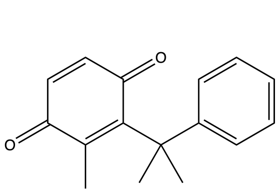	37	043	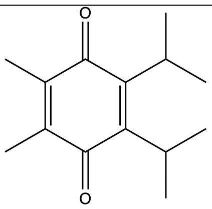	36

Molecule ID	Structure	Size	Molecule ID	Structure	Size
044	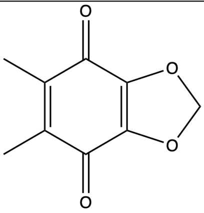	21	052	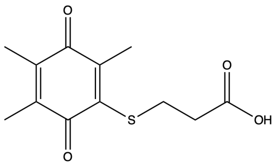	31
045	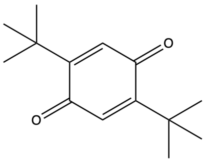	36	053	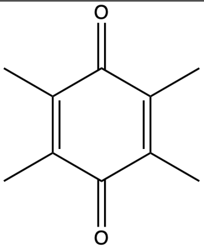	24
046	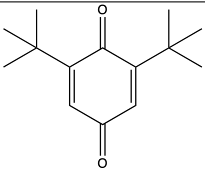	36	054	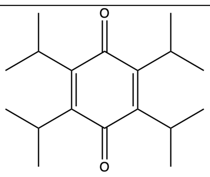	48
047	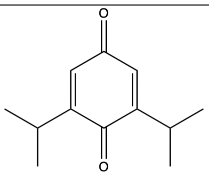	30	055	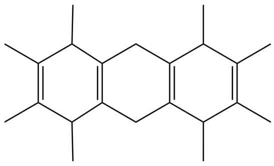	52
048	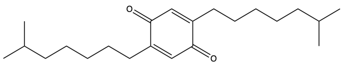	60	056	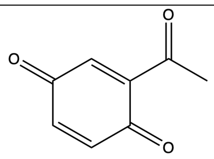	17
049	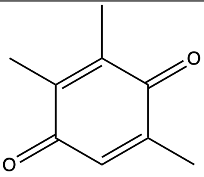	21	057	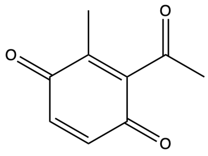	20
050	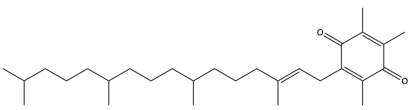	79	058	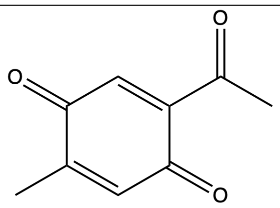	20
051	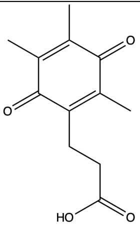	30	059	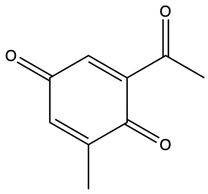	20

Molecule ID	Structure	Size	Molecule ID	Structure	Size
060	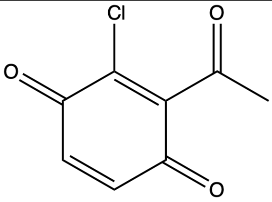	17	067	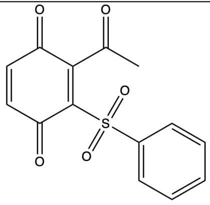	30
061	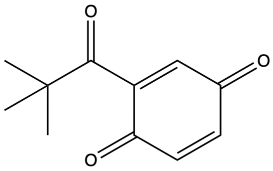	26	068	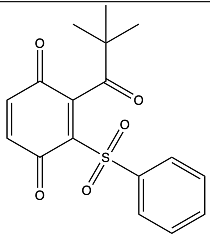	39
062	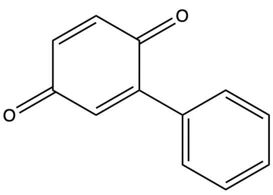	22	069	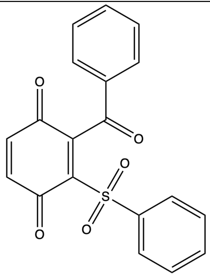	37
063	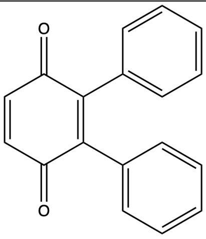	32	070	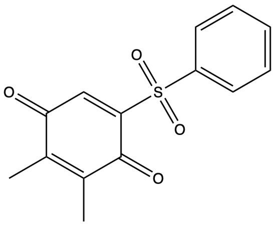	31
064	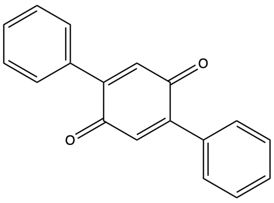	32	071	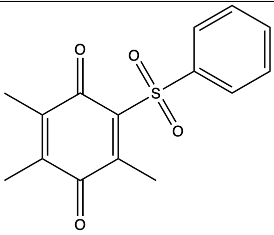	34
065	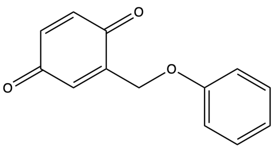	26	072	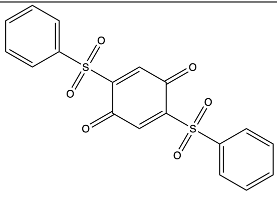	38
066	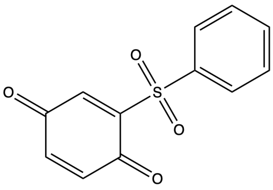	25	073	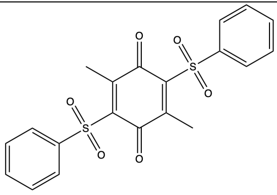	44

Molecule ID	Structure	Size	Molecule ID	Structure	Size
074	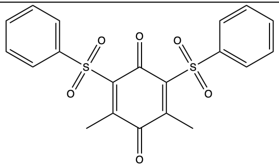	44	082	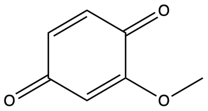	16
075	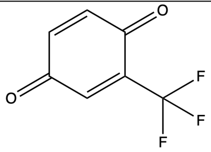	15	083	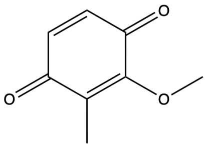	19
076	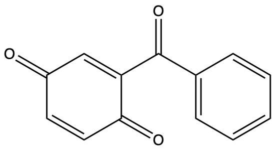	24	084	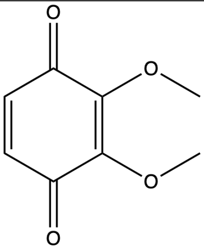	20
077	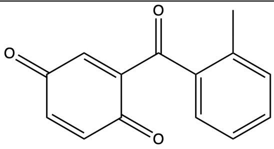	27	085	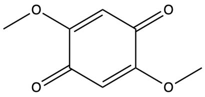	20
078	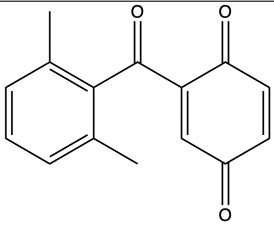	30	086	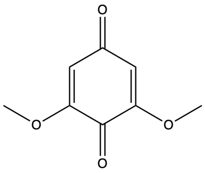	20
079	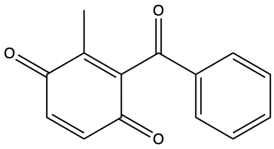	27	087	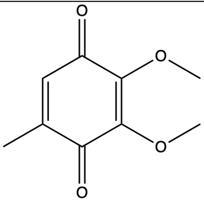	23
080	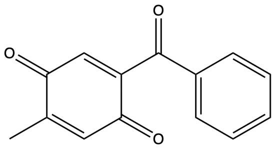	27	088	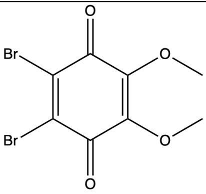	20
081	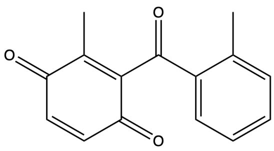	30	089	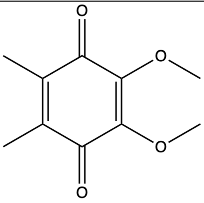	26

Molecule ID	Structure	Size	Molecule ID	Structure	Size
090	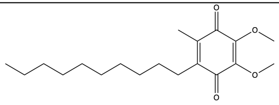	53	100	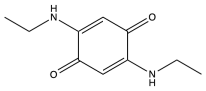	28
091	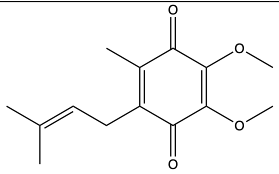	36	101	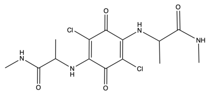	42
092	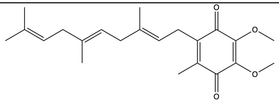	62	102	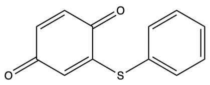	23
093	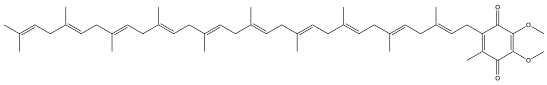	153	103	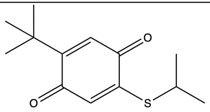	34
094	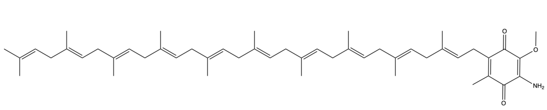	151	104	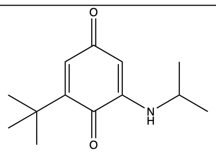	35
095	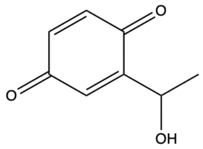	19	105	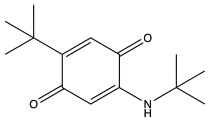	38
096	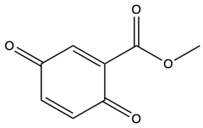	18	106	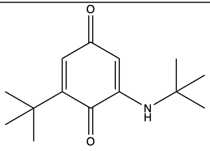	38
097	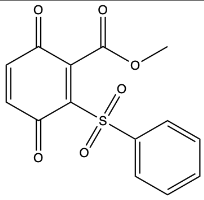	31	107	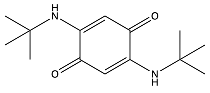	40
098	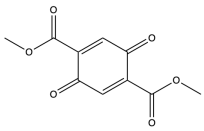	24	108	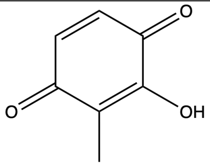	16
099	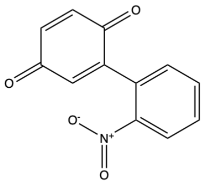	24	109	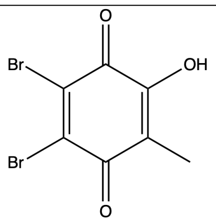	16

Molecule ID	Structure	Size	Molecule ID	Structure	Size
110	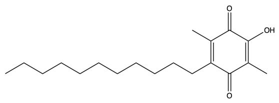	52	119	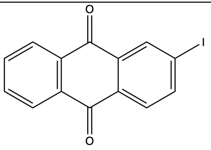	24
111	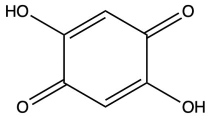	14	120	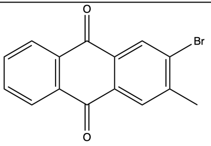	27
112	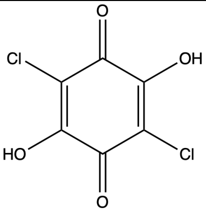	14	121	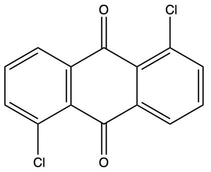	24
113	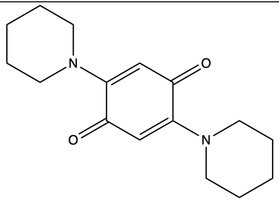	42	122	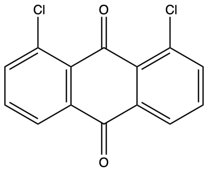	24
114	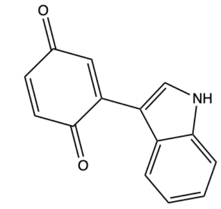	26	123	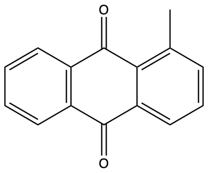	27
115	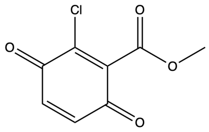	18	124	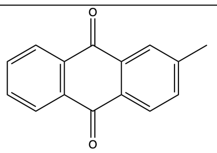	27
116	Undetermined	N/A	125	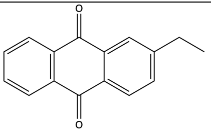	30
117	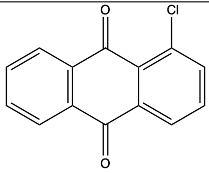	24	126	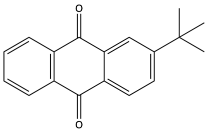	36
118	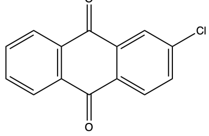	24	127	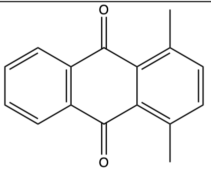	30

Molecule ID	Structure	Size	Molecule ID	Structure	Size
128	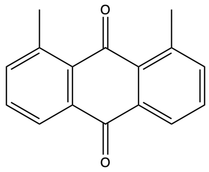	30	137	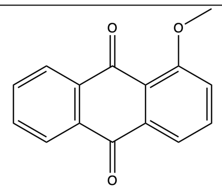	28
129	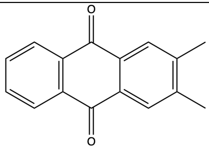	30	138	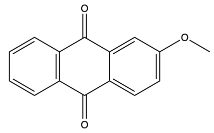	28
130	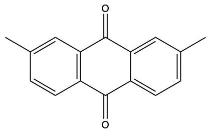	30	139	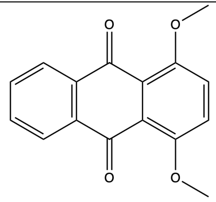	32
131	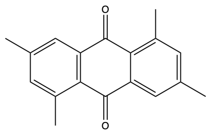	36	140	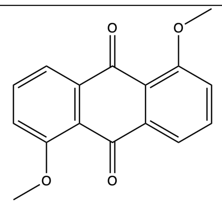	32
132	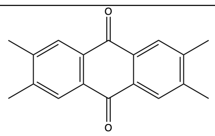	36	141	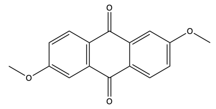	32
133	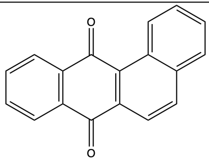	30	142	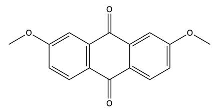	32
134	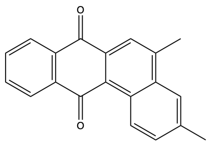	36	143	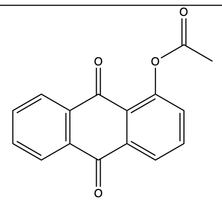	30
135	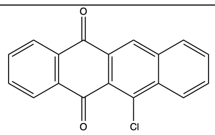	30	144	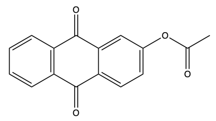	30
136	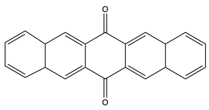	36	145	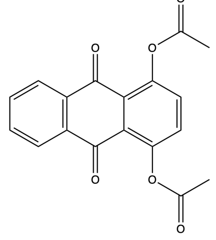	36

Molecule ID	Structure	Size	Molecule ID	Structure	Size
146	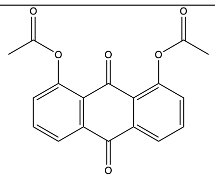	36	154	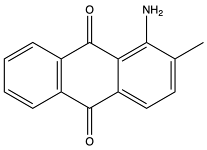	29
147	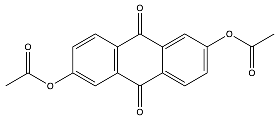	36	155	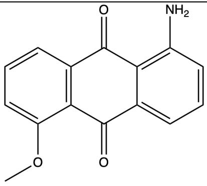	30
148	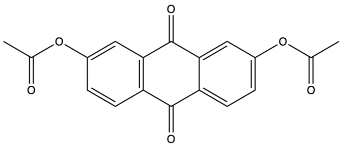	36	156	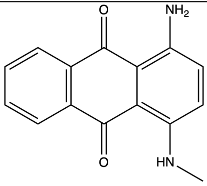	31
149	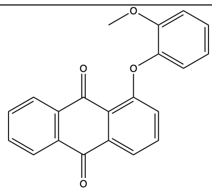	39	157	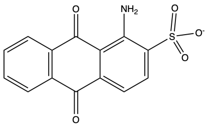	29
150	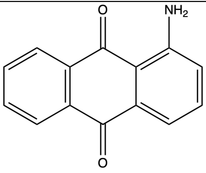	26	158	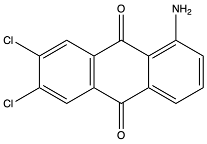	26
151	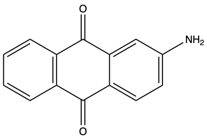	26	159	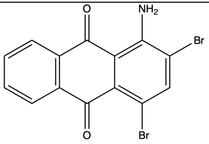	26
152	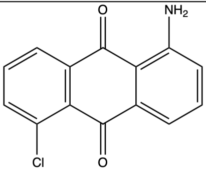	26	160	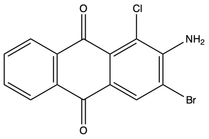	26
153	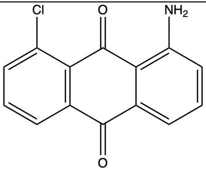	26	161	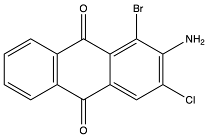	26

Molecule ID	Structure	Size	Molecule ID	Structure	Size
162	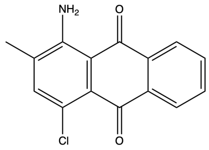	29	170	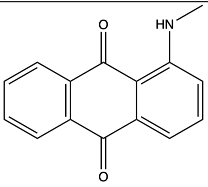	29
163	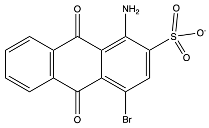	29	171	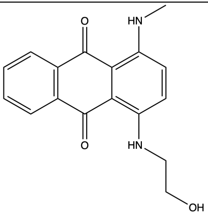	38
164	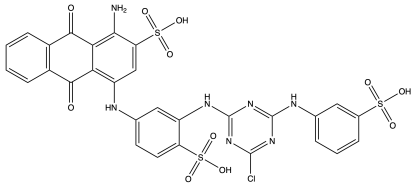	65	172	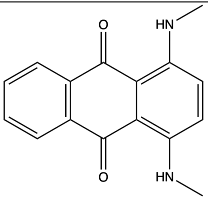	34
165	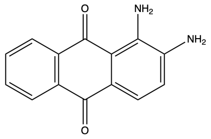	28	173	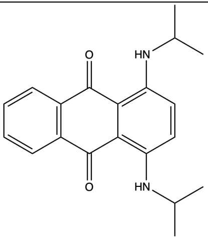	46
166	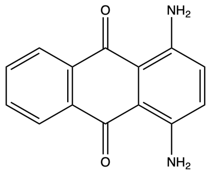	28	174	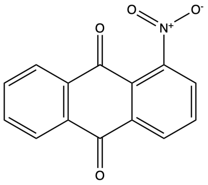	26
167	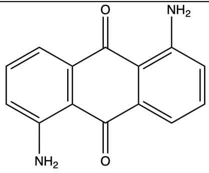	28	175	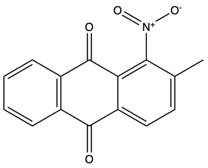	29
168	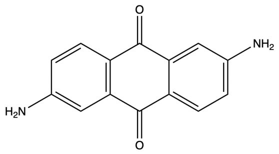	28	176	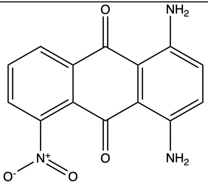	30
169	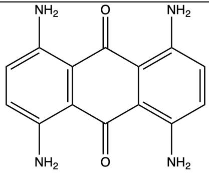	32	177	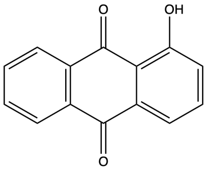	25

Molecule ID	Structure	Size	Molecule ID	Structure	Size
178	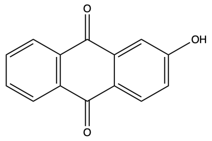	25	186	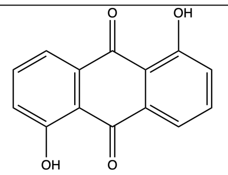	26
179	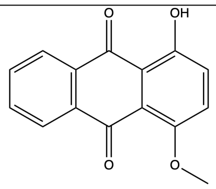	29	187	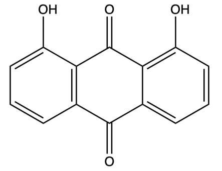	26
180	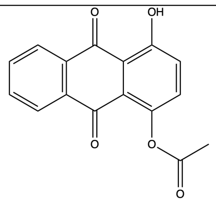	31	188	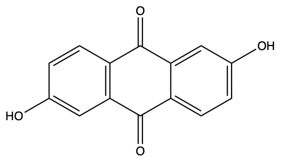	26
181	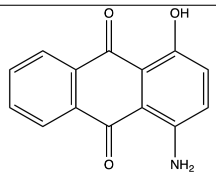	27	189	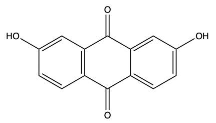	26
182	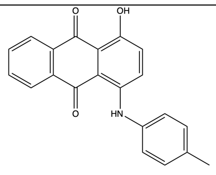	40	190	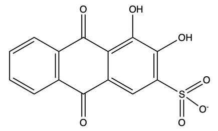	29
183	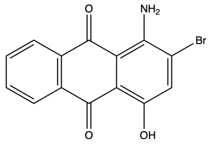	27	191	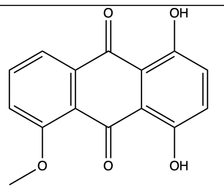	30
184	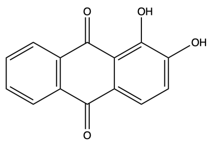	26	192	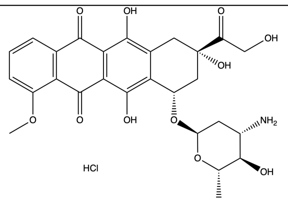	68
185	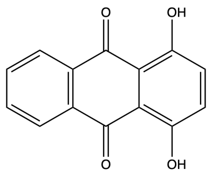	26	193	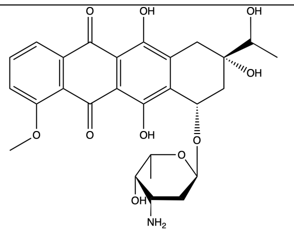	67

Molecule ID	Structure	Size	Molecule ID	Structure	Size
194	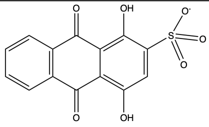	29	203	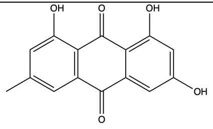	30
195	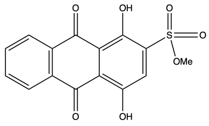	33	204	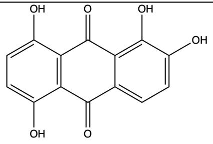	28
196	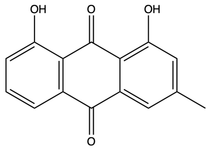	29	205	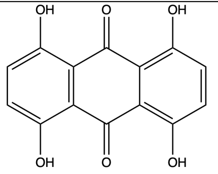	28
197	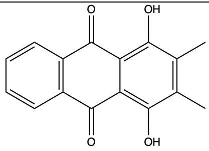	32	206	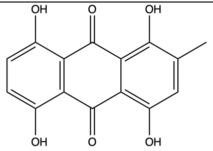	31
198	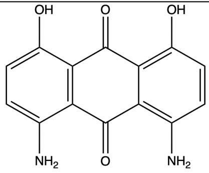	30	207	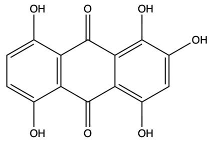	29
199	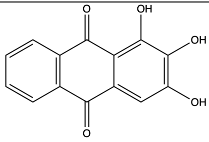	27	208	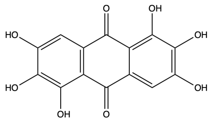	30
200	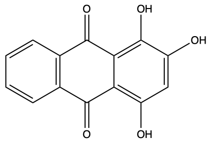	27	209	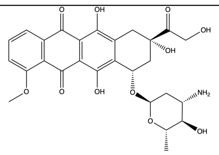	68
201	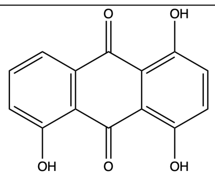	27	210	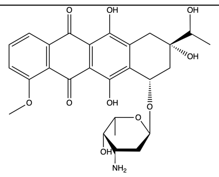	67
202	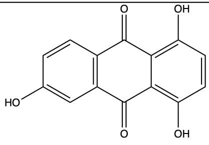	27	211	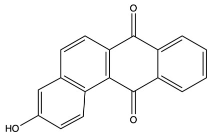	31

**Molecule ID**	**Structure**	**Size**	**Molecule ID**	**Structure**	**Size**
212	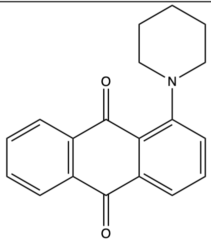	39	221	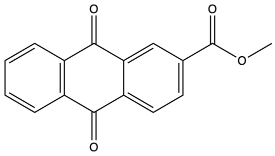	30
213	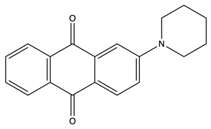	39	222	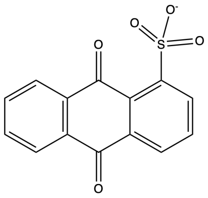	27
214	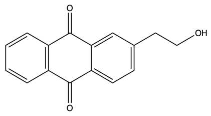	31	223	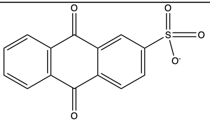	27
215	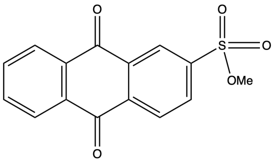	31	224	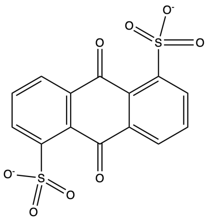	30
216	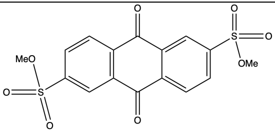	38	225	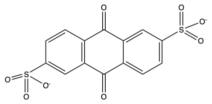	30
217	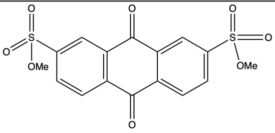	38	226	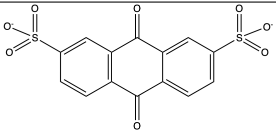	30
218	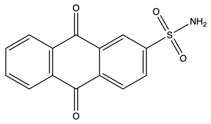	29	227	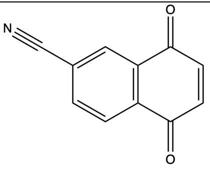	19
219	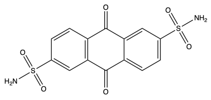	34	228	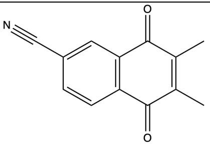	25
220	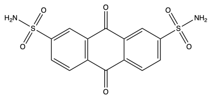	34	229	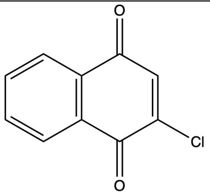	18

Molecule ID	Structure	Size	Molecule ID	Structure	Size
230	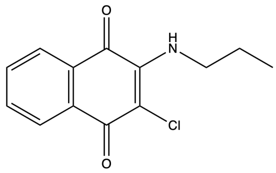	29	237	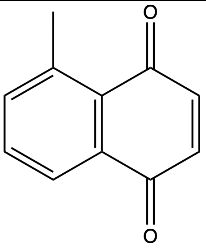	21
231	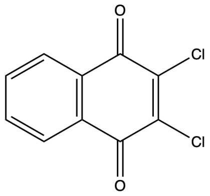	18	238	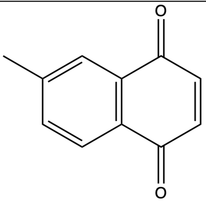	21
232	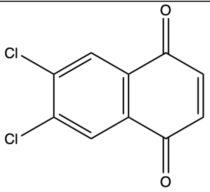	18	239	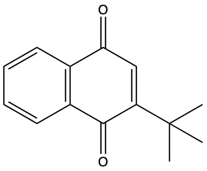	30
233	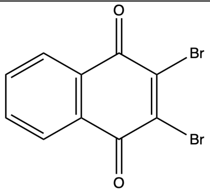	18	240	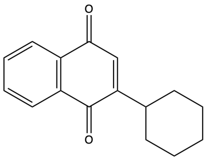	34
234	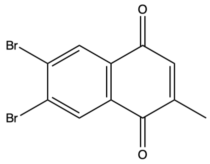	21	241	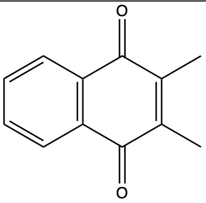	24
235	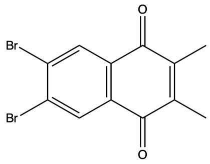	24	242	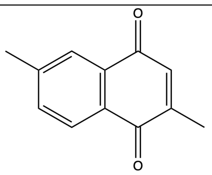	24
236	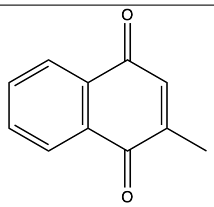	21	243	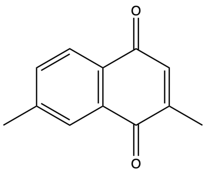	24

Molecule ID	Structure	Size	Molecule ID	Structure	Size
244	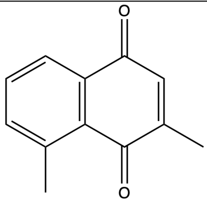	24	251	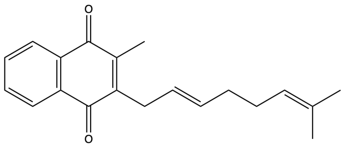	47
245	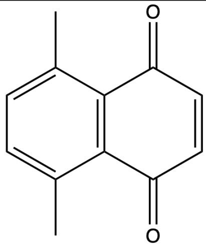	24	252	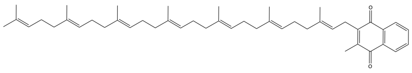	112
246	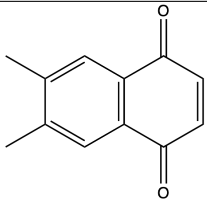	24	253	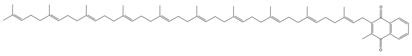	151
247	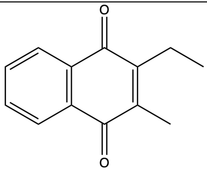	27	254	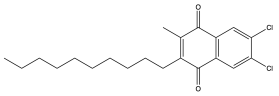	51
248	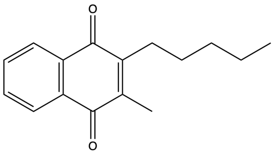	36	255	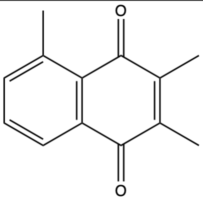	27
249	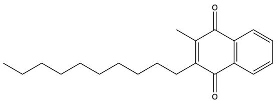	51	256	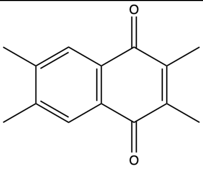	30
250	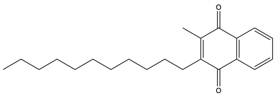	54	257	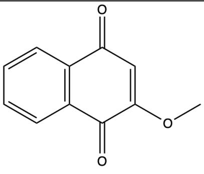	22

Molecule ID	Structure	Size	Molecule ID	Structure	Size
258	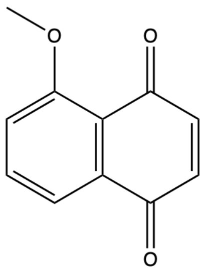	22	264	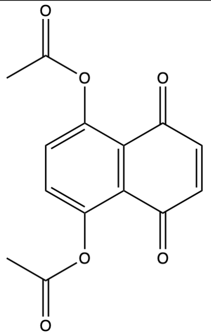	30
259	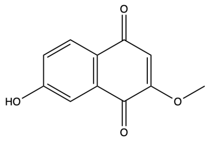	23	265	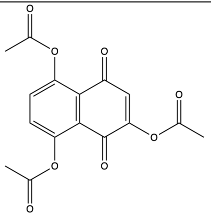	36
260	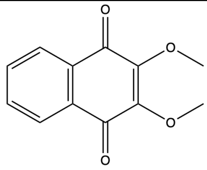	26	266	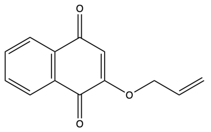	26
261	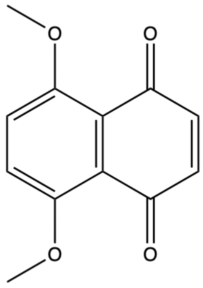	26	267	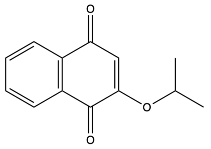	28
262	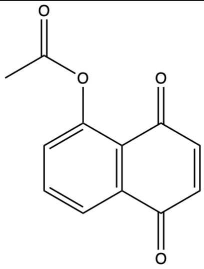	24	268	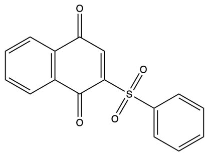	31
263	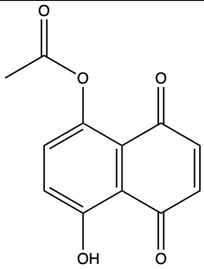	25	269	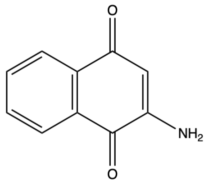	20

Molecule ID	Structure	Size	Molecule ID	Structure	Size
270	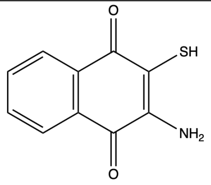	21	278	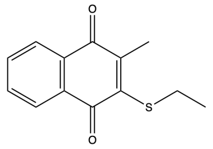	28
271	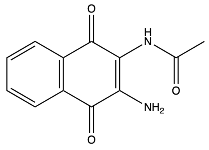	27	279	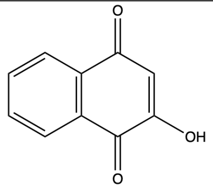	19
272	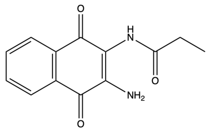	30	280	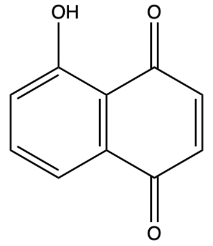	19
273	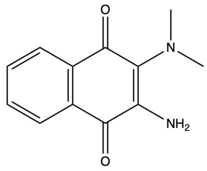	28	281	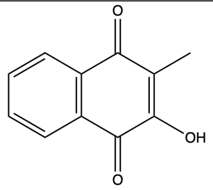	22
274	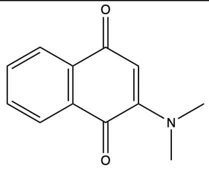	26	282	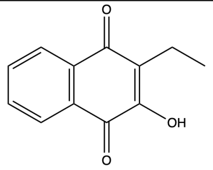	25
275	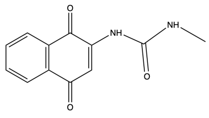	27	283	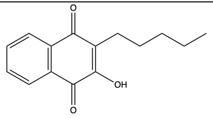	34
276	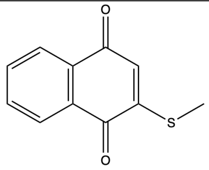	22	284	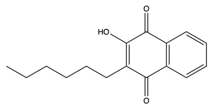	37
277	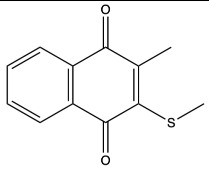	25	285	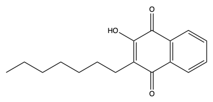	40

Molecule ID	Structure	Size	Molecule ID	Structure	Size
286	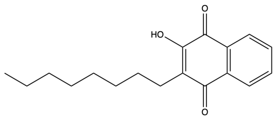	43	296	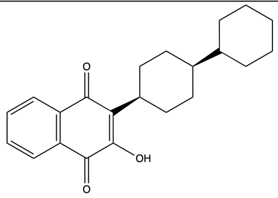	51
287	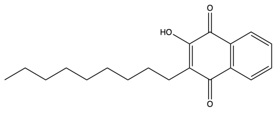	46	297	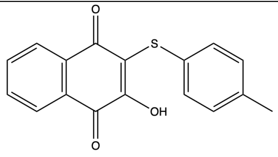	33
288	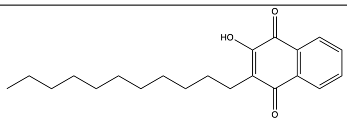	52	298	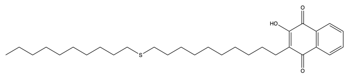	80
289	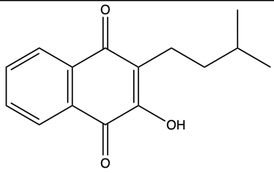	34	299	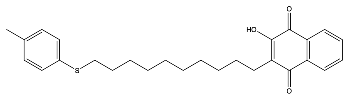	63
290	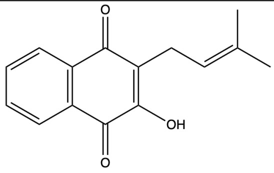	32	300	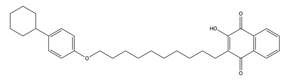	76
291	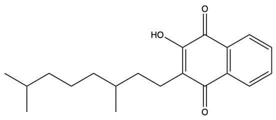	49	301	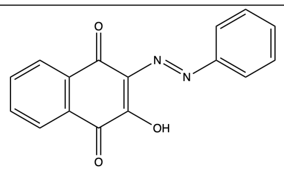	31
292	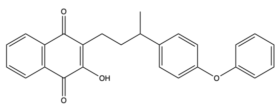	52	302	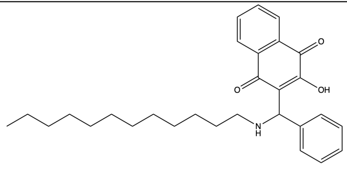	70
293	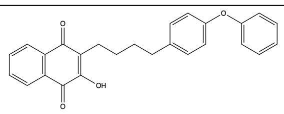	52	303	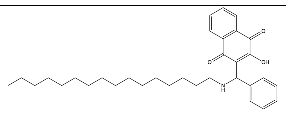	82
294	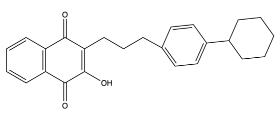	54	304	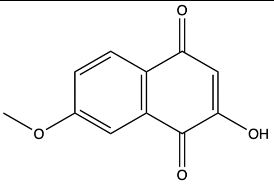	23
295	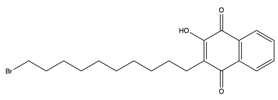	49	305	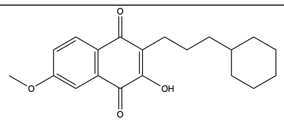	48

Molecule ID	Structure	Size	Molecule ID	Structure	Size
306	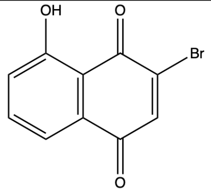	19	313	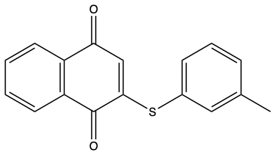	32
307	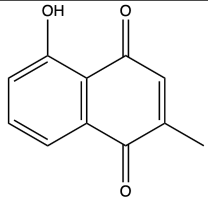	22	314	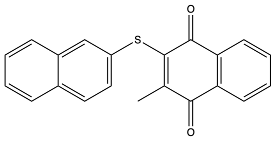	38
308	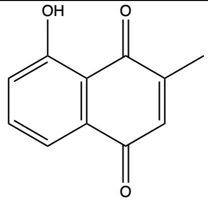	22	315	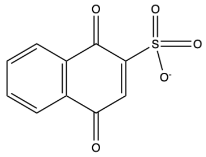	21
309	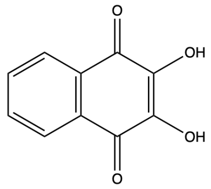	20	316	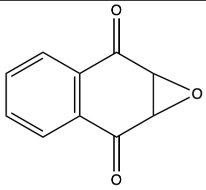	19
310	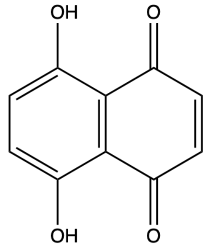	20	317	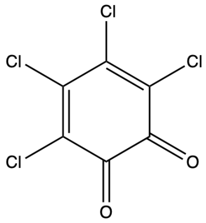	12
311	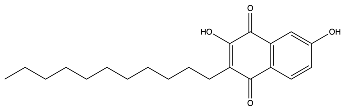	53	318	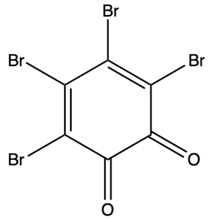	12
312	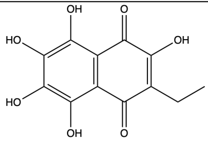	29	319	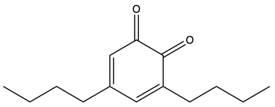	36

Molecule ID	Structure	Size	Molecule ID	Structure	Size
320	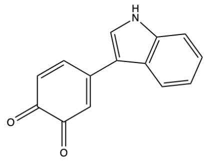	26	326	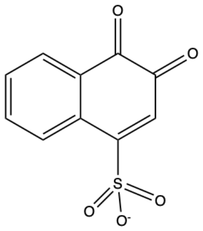	21
321	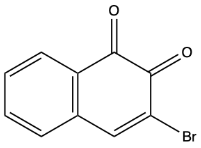	18	327	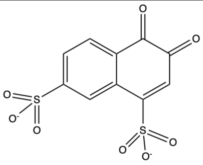	24
322	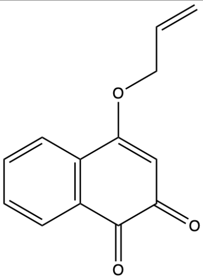	26	328	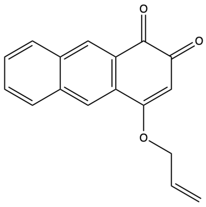	32
323	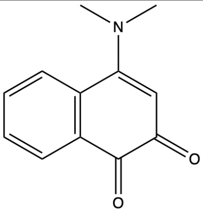	26	329	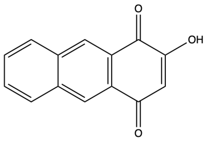	25
324	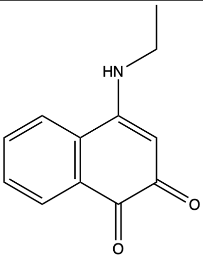	26	330	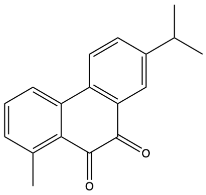	36
325	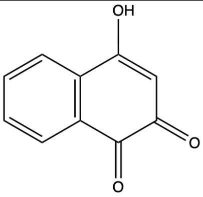	19	331	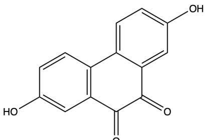	26

Molecule ID	Structure	Size	Molecule ID	Structure	Size
332	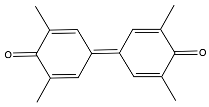	34	339	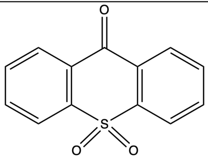	25
333	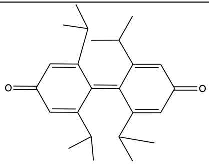	58	340	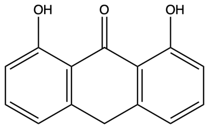	27
334	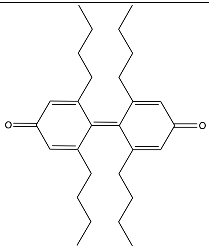	70	341	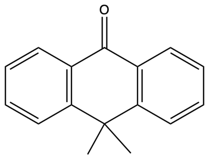	31
335	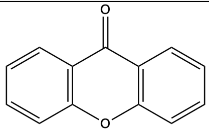	23	342	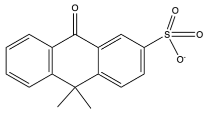	34
336	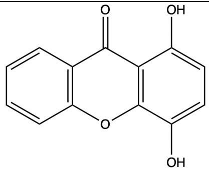	25	343	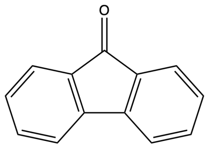	22
337	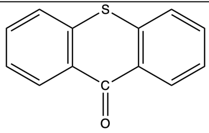	23	344	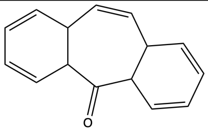	26
338	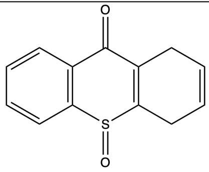	24	345	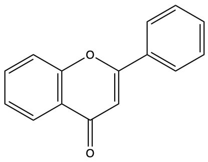	27

Molecule ID	Structure	Size
346	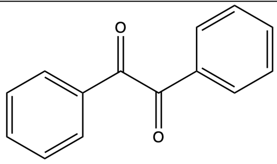	26
347	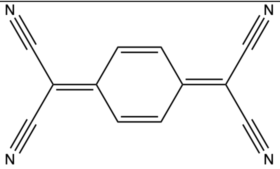	20
348	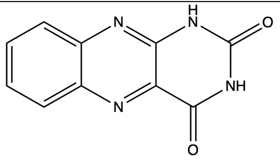	22
349	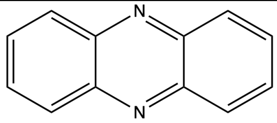	22

Table S1: List of 350 quinone derivative structures from tables 2, 5, 6, and 11 from the Prince et al. study (BBA-Bioenergetics, 1863(6), 2022, 148558). Each molecule is assigned a molecule ID and molecular size (i.e., number of atoms). As discussed in the main text, five molecules are excluded from our computational analysis; 094, 116, 164, 209, and 210. 117 molecules (000 to 116) are benzoquinone derivatives, out of which two are excluded, giving 115 BQ molecules in our benchmark. 110 molecules (117 to 226) are anthraquinone derivatives out of which three are excluded (164, 209, and 210), giving 107 AQ molecules in our benchmark. 90 molecules (227 to 316) are naphthoquinone derivatives, all of which were included in our NQ set. Lastly, 33 molecules are other miscellaneous quinones and aromatic compounds (317 to 349), all of which were included in our Other set. Therefore, the total number of structures studied in our analysis is 345.

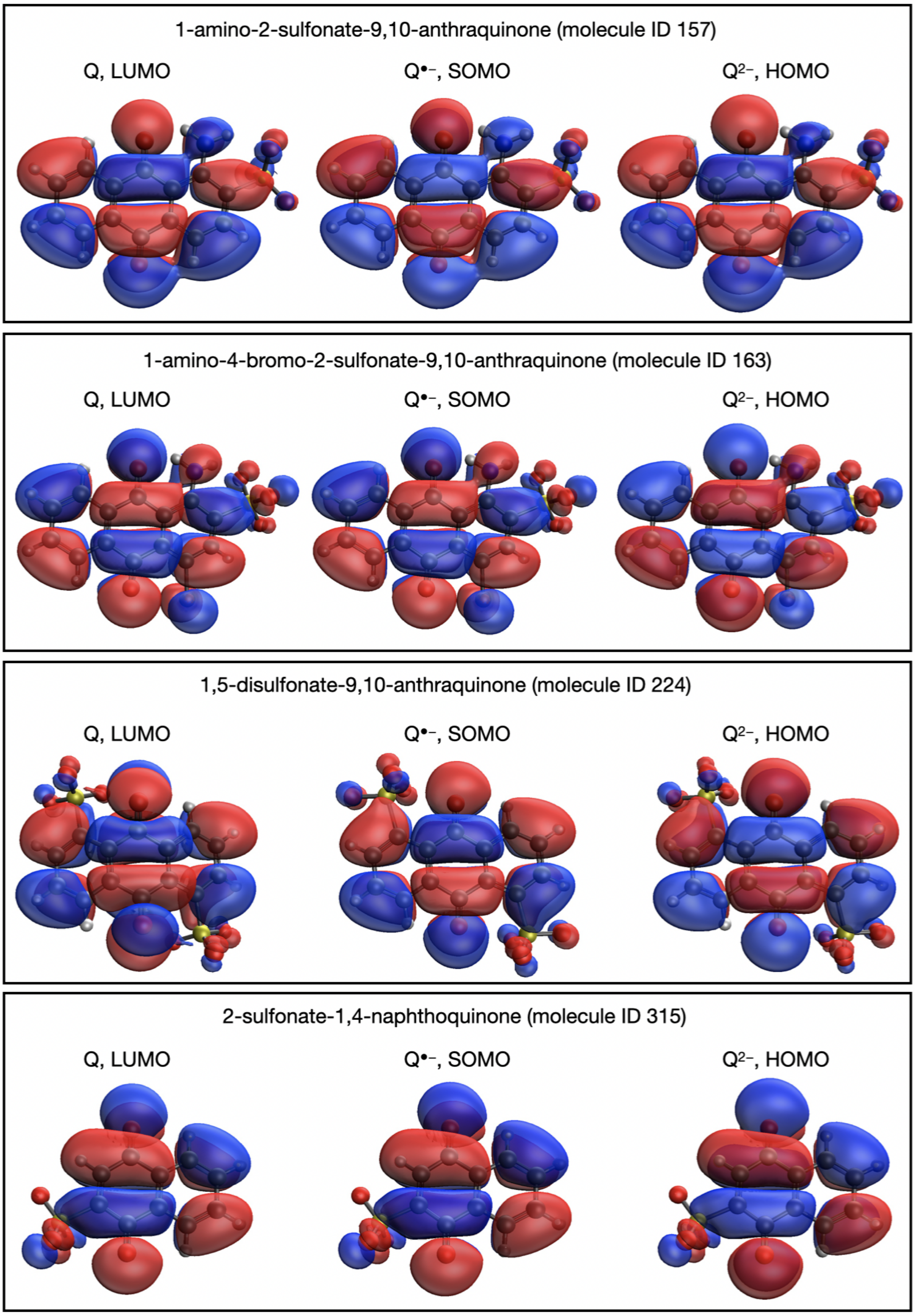

Figure S1: Figure showing the LUMO orbitals of Q, SOMO orbitals of $Q^{\bullet -}$ and HOMO orbitals of Q^{2-} for four sulfonate-containing molecules: 1-amino-2-sulfonate-9,10-anthraquinone (ID 157), 1-amino-4-bromo-2-sulfonate-9,10-anthraquinone (ID 163), 1,5-disulfonate-9,10-anthraquinone (ID 224), and 2-sulfonate-1,4-naphthoquinone (ID 315).

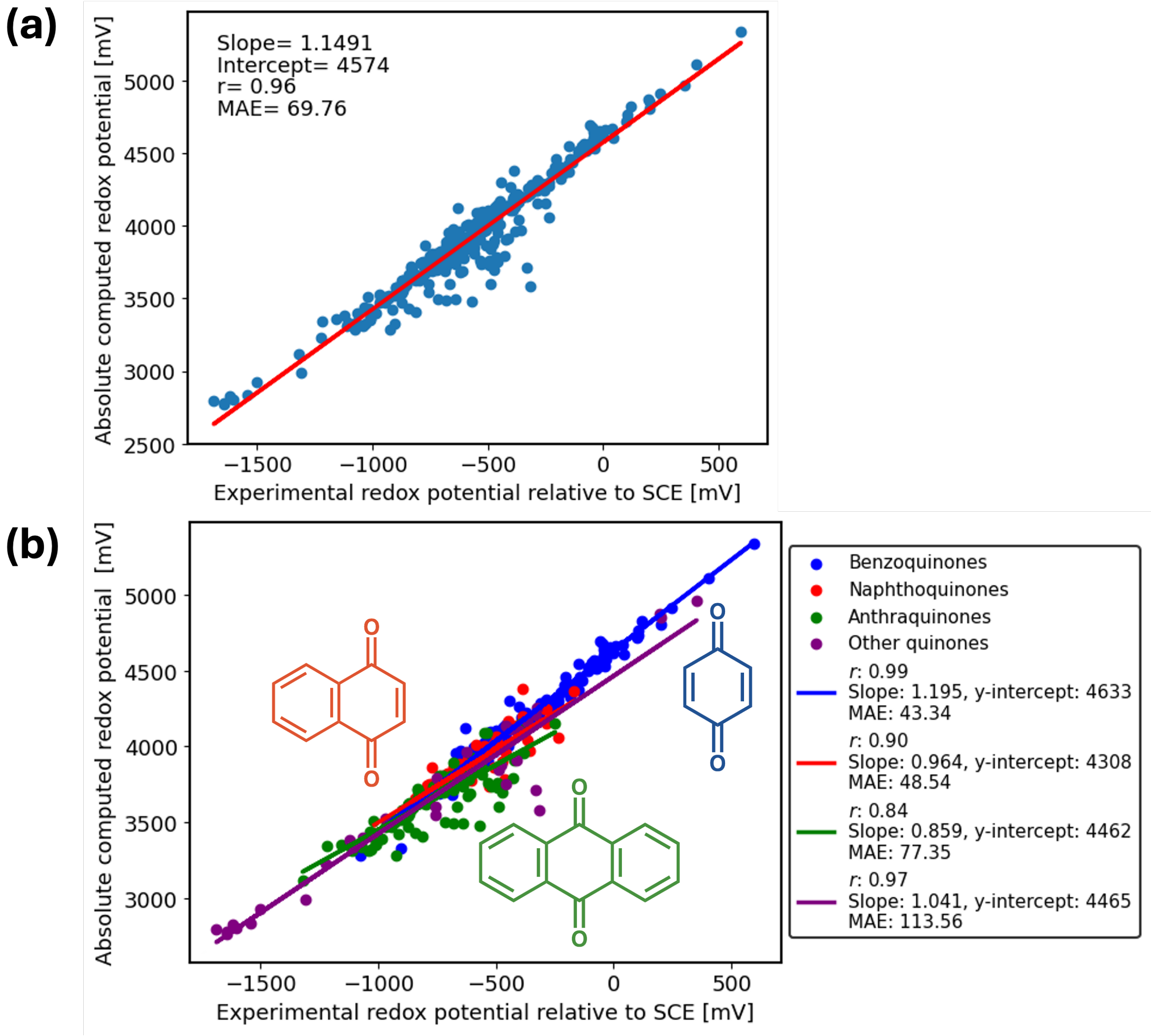

Figure S2: (a) Correlation between $Q/Q^{\bullet-}$ experimental reduction potentials relative to SCE and absolute calculated reduction potentials for N = 345 total studied quinone derivatives in DMF using the **IEF-PCM-cycle** approach, without constraining the slope to 1. (b) Correlation between experimental reduction potential and absolute calculated ones fitted separately for 4 groups without constraining the slope to 1: 115 Benzoquinones (blue), 90 Naphthoquinones (red), 107 Anthraquinones (green), and 33 Others (purple). Experimental data were obtained from Prince *et al.*

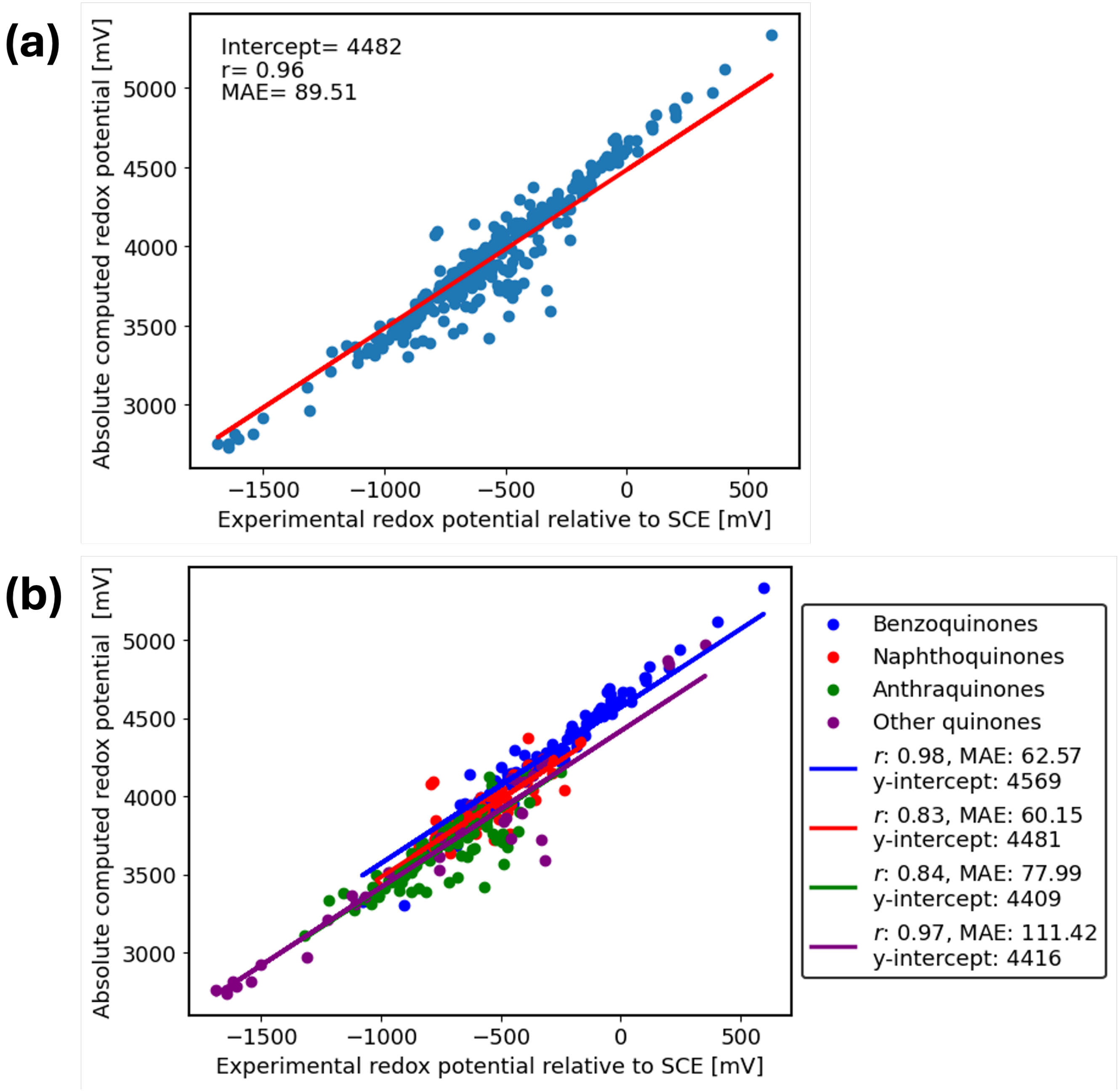

Figure S3: (a) Correlation between $Q/Q^{\bullet-}$ experimental reduction potentials relative to SCE and absolute calculated reduction potentials for N = 345 total studied quinone derivatives in DMF using **IEF-PCM-direct** solvation, without constraining the slope to 1. (b) Correlation between experimental reduction potential and absolute calculated ones fitted separately for 4 groups without constraining the slope to 1: 115 Benzoquinones (blue), 90 Naphthoquinones (red), 107 Anthraquinones (green), and 33 Others (purple). Experimental data were obtained from Prince *et al.*

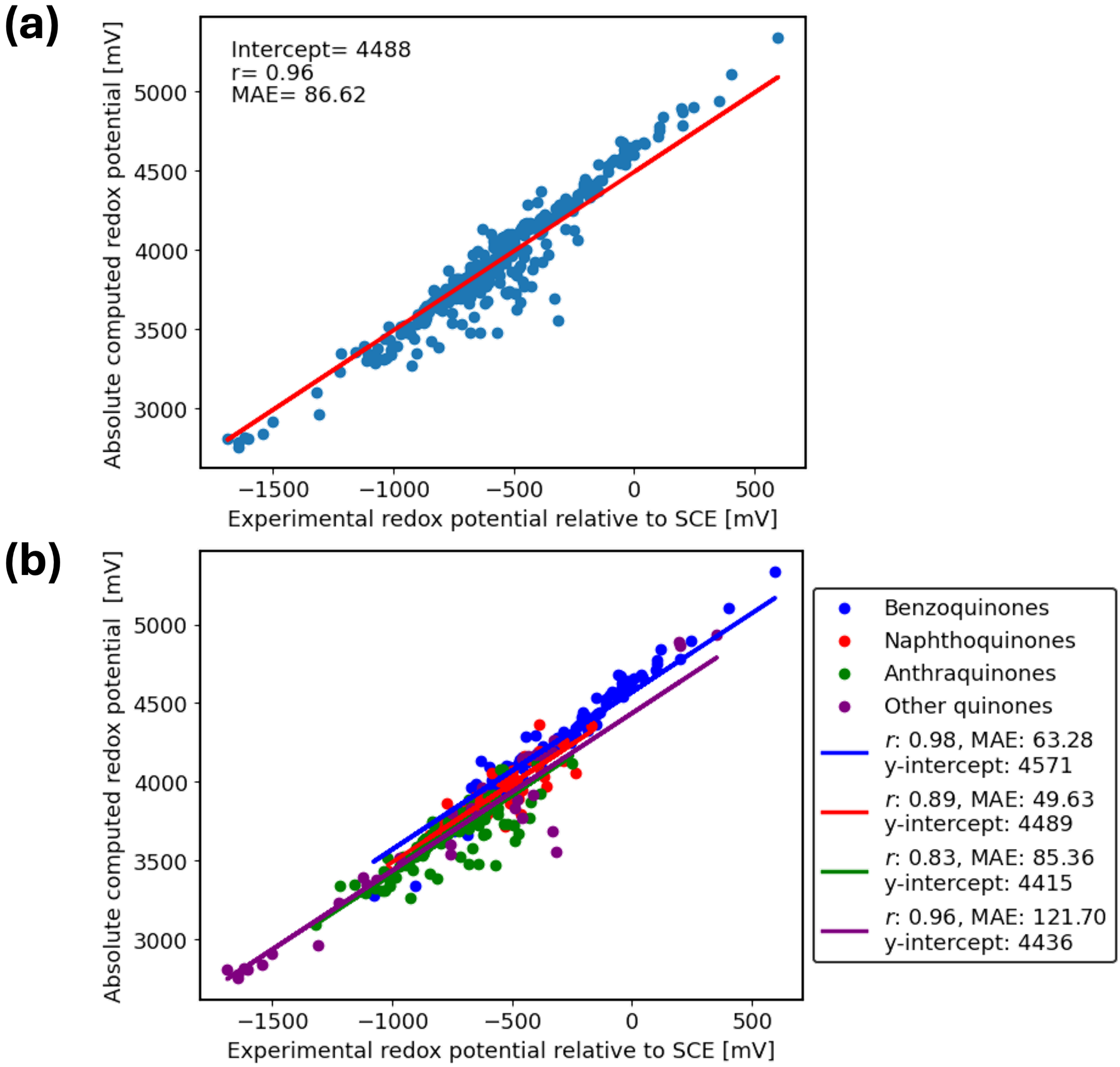

Figure S4: (a) Correlation between $Q/Q^{\bullet-}$ experimental reduction potentials relative to SCE and absolute calculated reduction potentials for N = 345 total studied quinone derivatives in DMF using **C-PCM** solvation, without constraining the slope to 1. (b) Correlation between experimental reduction potential and absolute calculated ones fitted separately for 4 groups without constraining the slope to 1: 115 Benzoquinones (blue), 90 Naphthoquinones (red), 107 Anthraquinones (green), and 33 Others (purple). Experimental data were obtained from Prince *et al.*

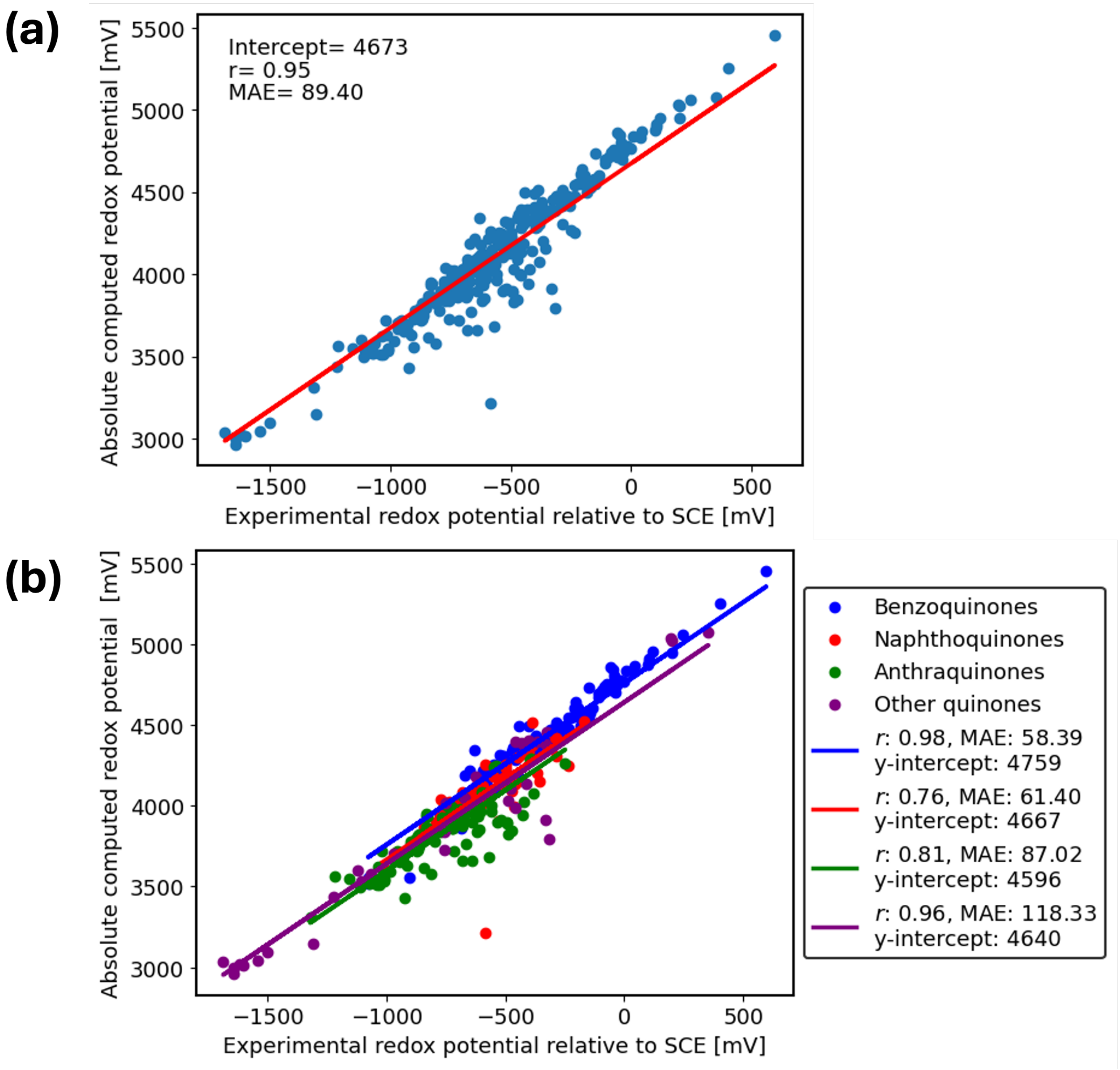

Figure S5: (a) Correlation between $Q/Q^{\bullet -}$ experimental reduction potentials relative to SCE and absolute calculated reduction potentials for N = 345 total studied quinone derivatives in DMF using **COSMO** solvation, without constraining the slope to 1. (b) Correlation between experimental reduction potential and absolute calculated ones fitted separately for 4 groups without constraining the slope to 1: 115 Benzoquinones (blue), 90 Naphthoquinones (red), 107 Anthraquinones (green), and 33 Others (purple). Experimental data were obtained from Prince *et al.*

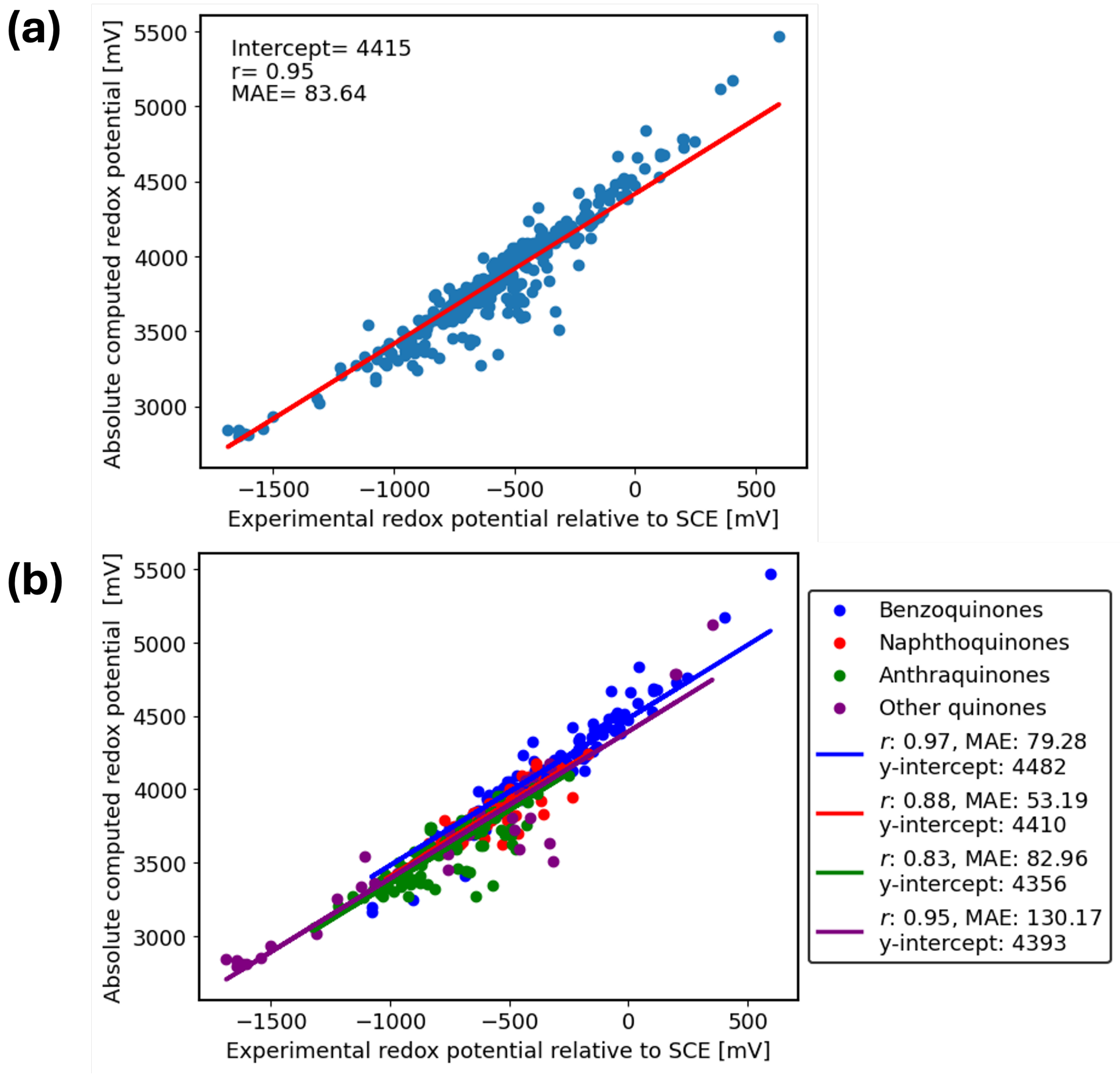

Figure S6: (a) Correlation between Q/Q$^{\bullet-}$ experimental reduction potentials relative to SCE and absolute calculated reduction potentials for N = 345 total studied quinone derivatives in DMF using **SM12** solvation, without constraining the slope to 1. (b) Correlation between experimental reduction potential and absolute calculated ones fitted separately for 4 groups without constraining the slope to 1: 115 Benzoquinones (blue), 90 Naphthoquinones (red), 107 Anthraquinones (green), and 33 Others (purple). Experimental data were obtained from Prince *et al.*

Table S2: MAEs for $Q/Q^{\bullet -}$ reduction potentials for molecules with titratable versus non-titratable subsituents using the PCM-cycle approach. Here we further divide molecules based on their substituents. N represents the total number of molecules having each type of substituent across all quinones, or across every parent subgroup.

**Substituents**	**N**	**MAEs for all quinones**	**MAEs for Benzo-quinones**	**MAEs for Naphtho-quinones**	**MAEs for Anthra-quinones**	**MAEs for other quinones**
Titratables	140	83.9	107.2	57.6	93.7	96.7
Non-titratables	205	55.7	55.9	41.3	55.6	125
Cyano	6	116.1	135.1	11.6	-	178.0
Sulfonates	13	113.2	-	107.3	124.5	81.1
Acetoxy	12	89.2	49.6	137.5	67.3	-
Amine	53	85.9	118.1	30.4	92.6	92.7
Hydroxy	81	83.3	69.4	63.1	104.6	101.2
Fluorine	3	79.6	109.8	-	-	19.0
Oxy	127	70.9	57.6	60.8	81.7	115.1
Chlorine	33	59.6	58.1	80.8	50.3	241.8
Bromine	28	58.8	38.3	71.0	58.4	183.6
Nitro	5	42.9	34.7	-	48.5	-
Thio	14	39.9	35.0	35.8	-	66.1
Iodine	6	38.7	38.6	-	39.0	-

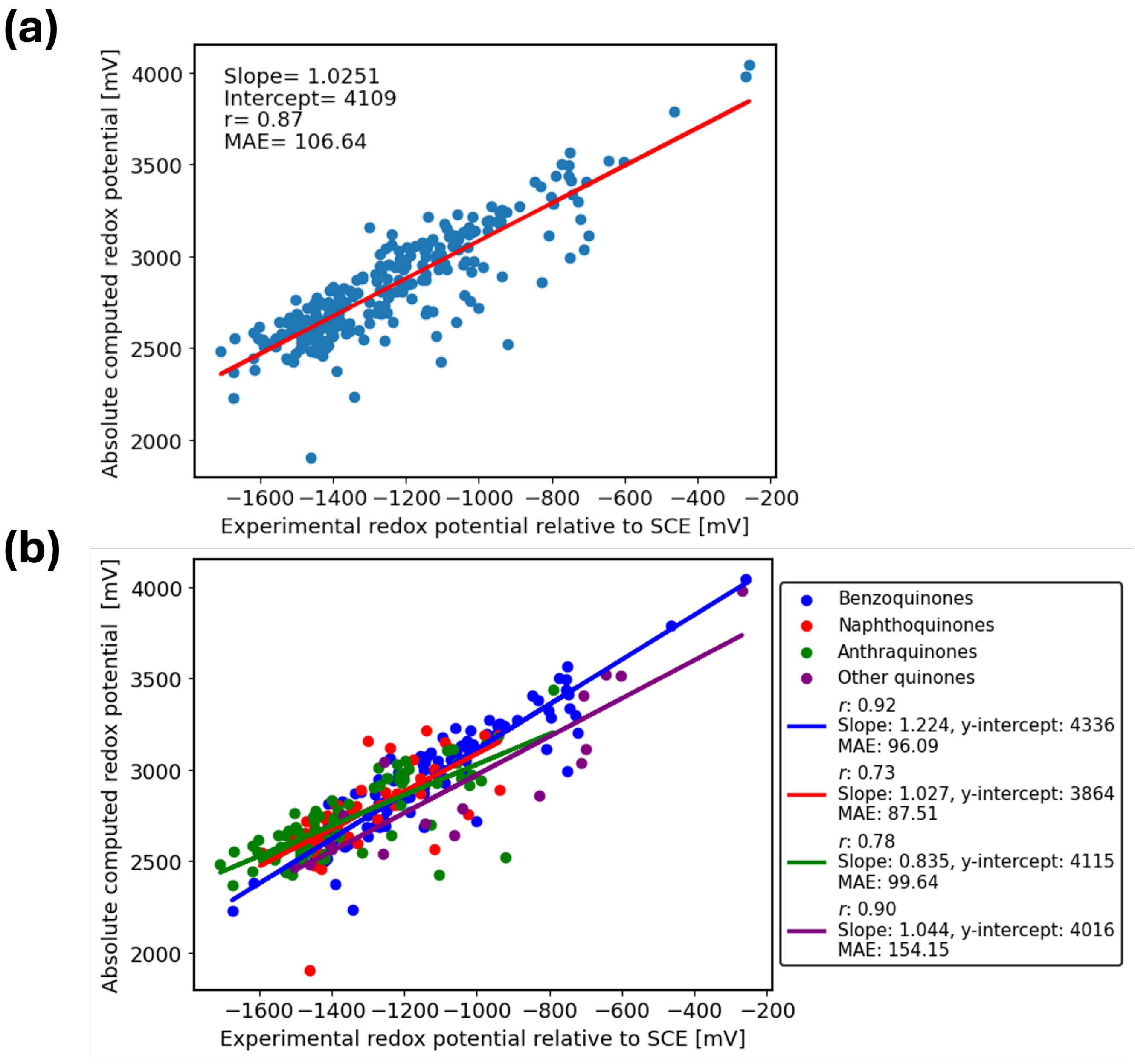

Figure S7: (a) Correlation between $Q^{\bullet-}/Q^{2-}$ experimental reduction potentials relative to SCE and absolute calculated reduction potentials for N = 265 total studied quinone derivatives in DMF using **IEF-PCM-cycle** solvation when the slope is not constrained to 1. (b) Correlation between experimental reduction potentials and absolute calculated ones fitted separately for 4 groups: 89 Benzoquinones (blue), 75 Naphthoquinones (red), 83 Anthraquinones (green), and 18 Others (purple). Experimental data were obtained from Prince *et al.*

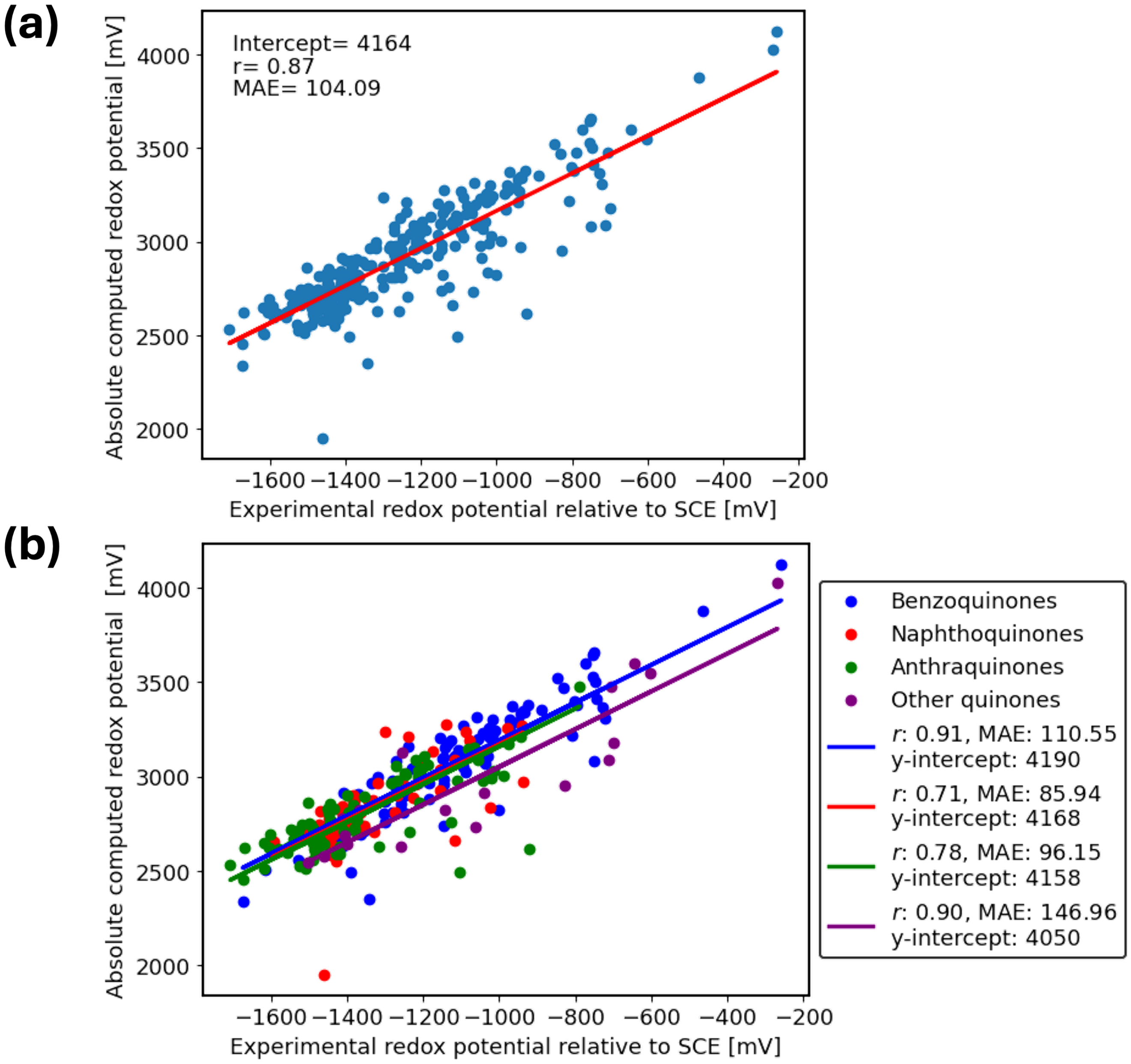

Figure S8: (a) Correlation between $Q^{\bullet-}/Q^{2-}$ experimental reduction potentials relative to SCE and absolute calculated reduction potentials for N = 265 total studied quinone derivatives in DMF using **C-PCM** solvation. (b) Correlation between experimental reduction potentials and absolute calculated ones fitted separately for 4 groups: 89 Benzoquinones (blue), 75 Naphthoquinones (red), 83 Anthraquinones (green), and 18 Others (purple). Experimental data were obtained from Prince *et al.*

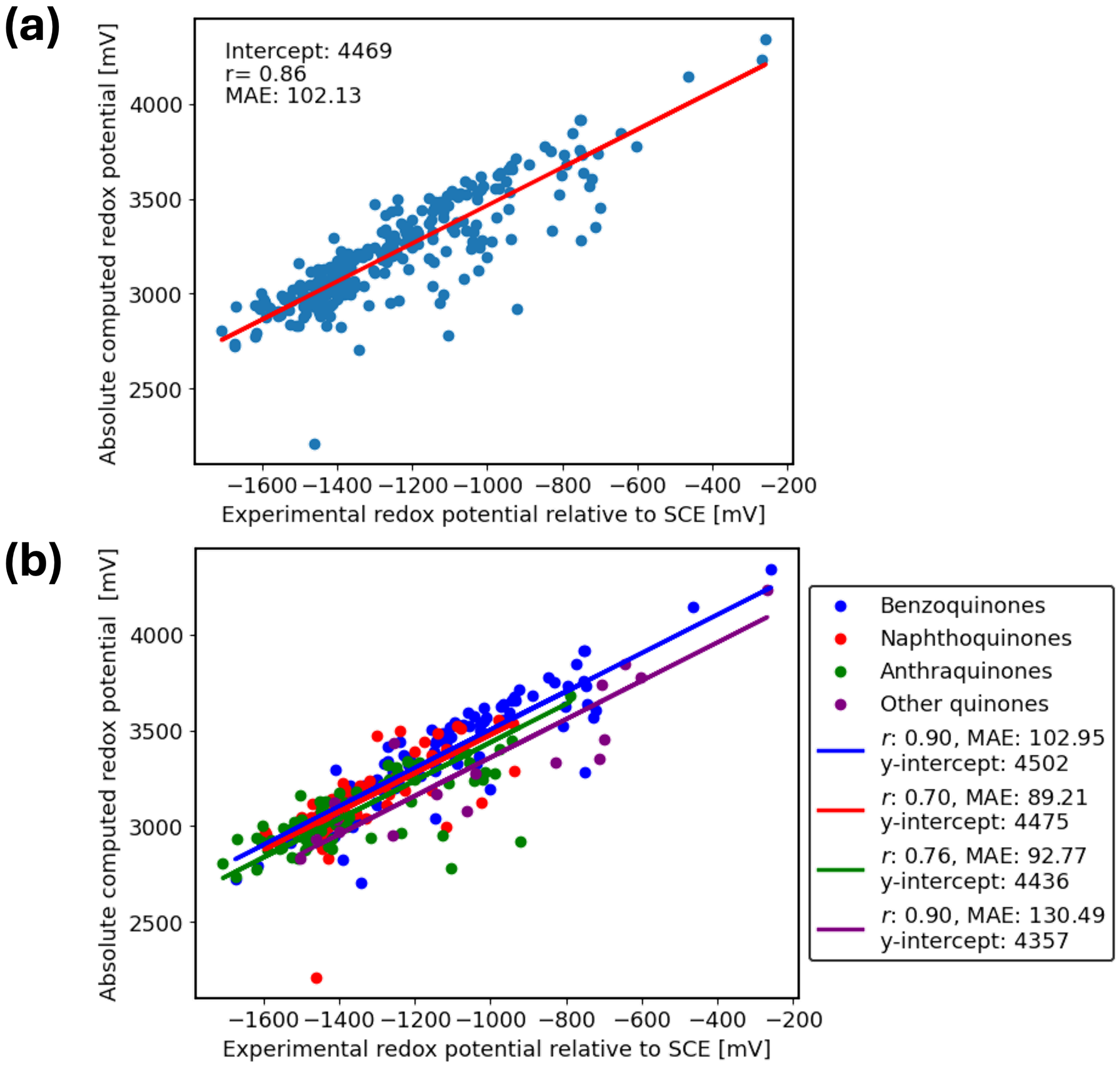

Figure S9: (a) Correlation between $Q^{\bullet -}/Q^{2-}$ experimental reduction potentials relative to SCE and absolute calculated reduction potentials for N = 265 total studied quinone derivatives in DMF using **COSMO** solvation. (b) Correlation between experimental reduction potentials and absolute calculated ones fitted separately for 4 groups: 89 Benzoquinones (blue), 75 Naphthoquinones (red), 83 Anthraquinones (green), and 18 Others (purple). Experimental data were obtained from Prince *et al.*

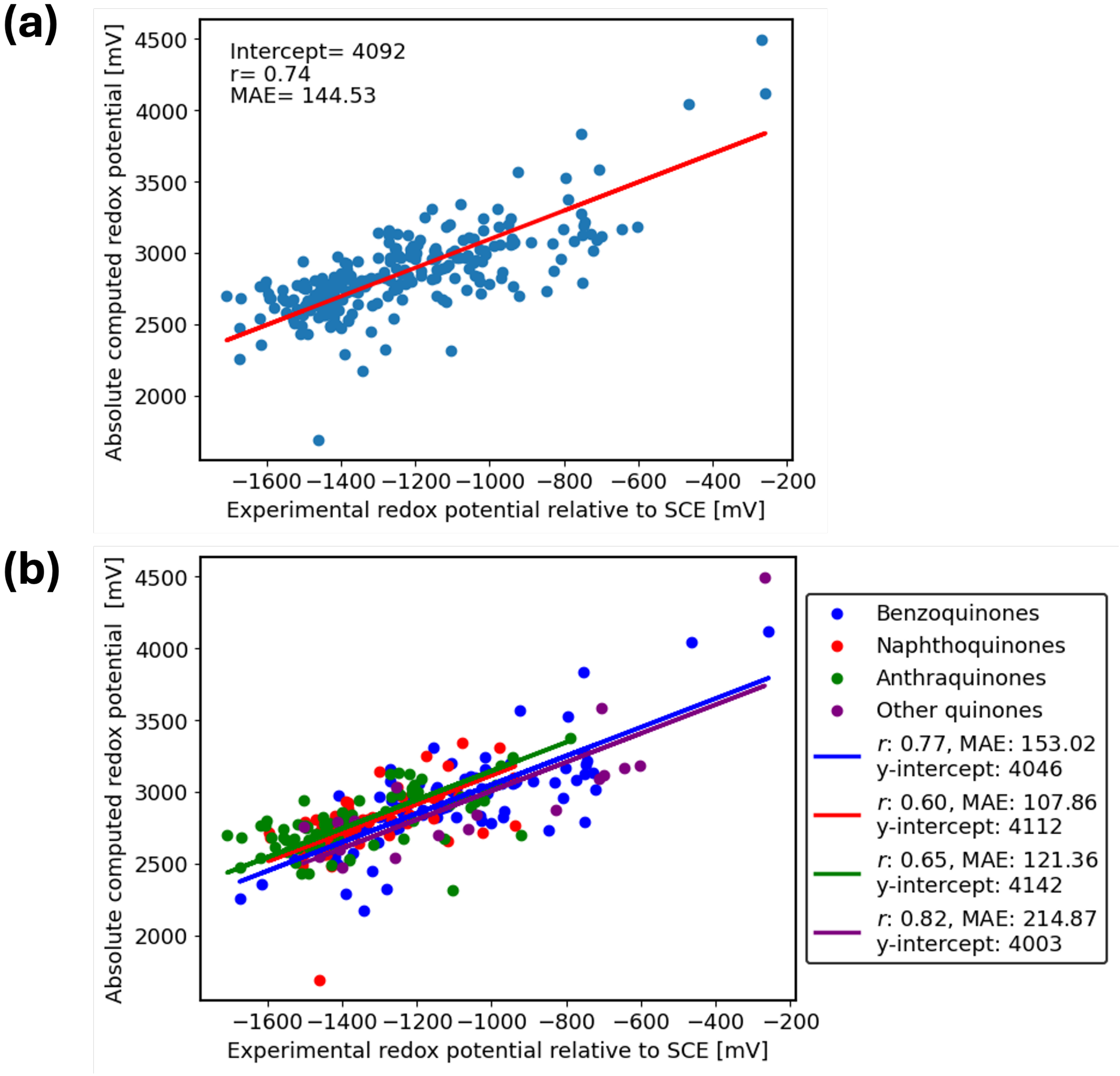

Figure S10: (a) Correlation between $Q^{\bullet-}/Q^{2-}$ experimental reduction potentials relative to SCE and absolute calculated reduction potentials for N = 265 total studied quinone derivatives in DMF using **SM12** solvation. (b) Correlation between experimental reduction potentials and absolute calculated ones fitted separately for 4 groups: 89 Benzoquinones (blue), 75 Naphthoquinones (red), 83 Anthraquinones (green), and 18 Others (purple). Experimental data were obtained from Prince *et al.*
